# Developing MXenes from Wireless Communication to Electromagnetic Attenuation

**DOI:** 10.1007/s40820-021-00645-z

**Published:** 2021-04-27

**Authors:** Peng He, Mao-Sheng Cao, Wen-Qiang Cao, Jie Yuan

**Affiliations:** 1 School of Materials Science and Engineering, Beijing Institute of Technology, Beijing, 100081 People’s Republic of China; 2 School of Information Engineering, Minzu University of China, Beijing, 100081 People’s Republic of China

**Keywords:** MXene, Wireless communication, Electromagnetic wave absorption, Electromagnetic interference shielding

## Abstract

There is an urgent global need for wireless communication utilizing materials that can provide simultaneous flexibility and high conductivity. Avoiding the harmful effects of electromagnetic (EM) radiation from wireless communication is a persistent research hot spot. Two-dimensional (2D) materials are the preferred choice as wireless communication and EM attenuation materials as they are lightweight with high aspect ratios and possess distinguished electronic properties. MXenes, as a novel family of 2D materials, have shown excellent properties in various fields, owing to their excellent electrical conductivity, mechanical stability, high flexibility, and ease of processability. To date, research on the utility of MXenes for wireless communication has been actively pursued. Moreover, MXenes have become the leading materials for EM attenuation. Herein, we systematically review the recent advances in MXene-based materials with different structural designs for wireless communication, electromagnetic interference (EMI) shielding, and EM wave absorption. The relationship governing the structural design and the effectiveness for wireless communication, EMI shielding, and EM wave absorption is clearly revealed. Furthermore, our review mainly focuses on future challenges and guidelines for designing MXene-based materials for industrial application and foundational research.

## Highlights

- The industrial application and foundational research of MXenes at gigahertz frequency are systematically reviewed.
- The design principles of “lightweight, wide, and strong” are specifically highlighted.
- Current challenges and future directions for MXenes in wireless communication and electromagnetic attenuation are outlined.

## Graphical abstract

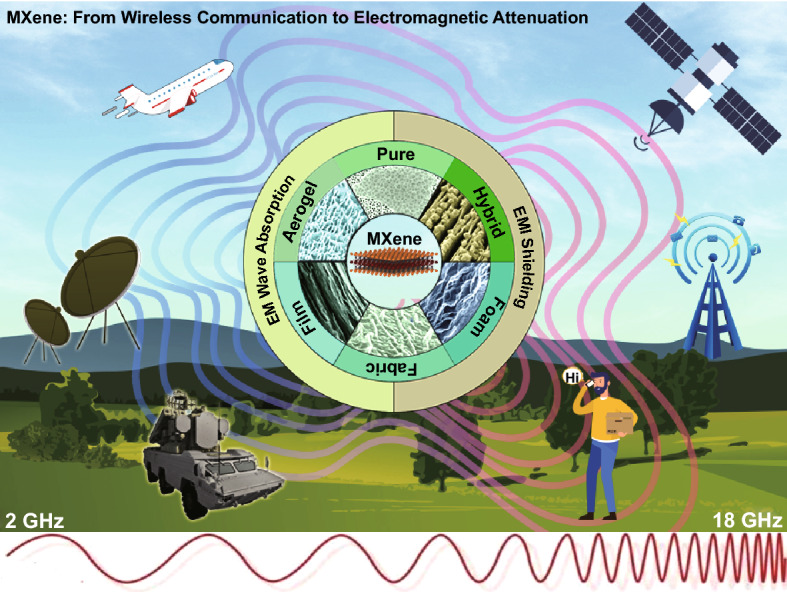

## Introduction

Wireless communication has been gaining popularity with the arrival of the age of artificial intelligence [1–3]. This is accompanied by a surge in the demand for all kinds of portable devices. These devices require a concealed integration of radio communication electronics without sacrificing lightweight and transportability [4–6]. Therefore, it is necessary to develop new routes of antenna fabrication [7, 8]. It is difficult to fabricate ultra-thin, flexible, and conformal antenna using traditional metal materials because of the skin depth limitation [9]. To overcome this shortcoming, carbon-based nanomaterials have been explored for wireless communication applications. For example, Elwi et al. prepared multi-walled carbon nanotube antennas that afforded a remarkable enhancement in the bandwidth [6]. Vacirca et al. reported an onion-like carbon antenna that showed a peak gain of − 1.48 dBi, just 3 dB less than that of a copper dipole antenna [8]. However, the low conductivity of carbon inhibits these materials from achieving reasonable radio-frequency performance. Thereafter, two-dimensional (2D) nanomaterials, such as graphene, MoS_2_, and others, have been used to fabricate antennas, thereby furnishing antennas with thinner patches. For example, Shin et al. fabricated a graphene antenna, affording a high transmitted power efficiency of 96.7% [1]. To date, it is still a challenge to find a flexible material with high conductivity for antenna fabrication.

On the other hand, the use of a large number of wireless communication devices will lead to an explosive increase in electromagnetic (EM) radiation on the scale of “big data” [10–12]. The undesired EM radiation directly affects the operation of electronic equipment and also indirectly influences human health, as long-term exposure to EM radiation may cause cancer and other health problems [13–16]. How to avoid the harm from EM radiation has always been a research hot spot. The development of electromagnetic interference (EMI) shielding and EM wave absorbing materials is the key to solving the above problem [17–22]. Recently, various materials have been globally studied as EMI shielding or/and EM wave absorbing materials, including zero-dimensional (0D), one-dimensional (1D), and 2D materials. Among them, 2D materials are the materials of choice as they are lightweight, have large aspect ratios, and offer distinguished electronic properties. For example, Cao’s group reported that chemically graphitized r-GOs exhibited high-efficiency EMI shielding effectiveness (EMI SE) at elevated temperatures. The EMI SE of the composites with 20 wt% r-GOs reached a maximum at ~ 38 dB [12]. Zhang et al. prepared 2D WS_2_-rGO heterostructure nanosheets. The composite containing 40 wt% WS_2_-rGO showed a minimum reflection loss (*RL*) of − 41.5 dB, with the absorption bandwidth reaching up to 13.62 GHz [15].

**Fig. 1:**
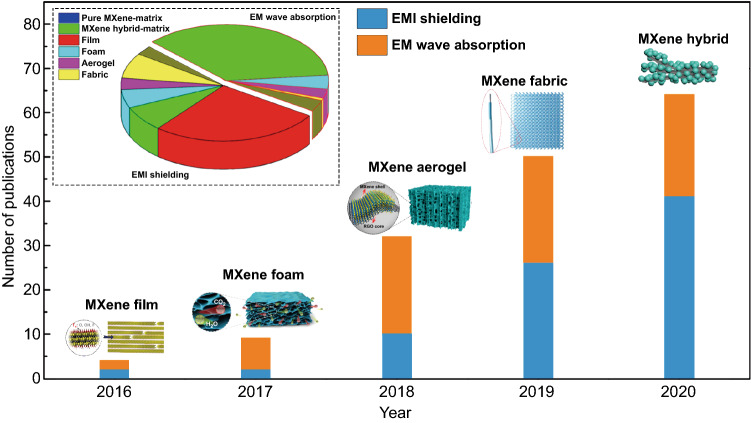
Number of publications focused on MXenes for EMI shielding and EM wave absorption. *Source*: Web of Science Core Collection, 2020 (inside: 2D MXenes serve as EMI shielding materials and EM wave absorption materials)

MXenes, as a novel family of 2D materials, possess huge potential in the fields of wireless communication, EMI shielding, and EM wave absorption owing to their excellent electrical conductivity, numerous family members, mechanical stability, high flexibility, and ease of processability [23, 24]. At present, research on MXene antennas is still in the exploratory stage, but the excellent properties of these materials for wireless communication have been widely regarded. Moreover, since the discovery of the outstanding EMI shielding performance of Ti_3_C_2_T_*x*_ MXene in 2016, MXenes have become the leading materials for EMI shielding and EM wave absorption with the fastest growing number of related research publications (Fig. 1). Moreover, as EM attenuation materials, the excellent chemical and physical properties of MXenes have facilitated the development of pure MXenes and hybrids with controlled structural designs such as films, foams, aerogels, and fabrics (inset of Fig. 1).

This review systematically summarizes the effects of MXenes with various structural designs on wireless communication, EMI shielding, and EM wave absorption. The most feasible strategies for high-performance wireless communication, EMI shielding, and EM wave absorption are revealed by discussing the different approaches for modifying the structures of MXenes. Finally, we provide an overview of the further development and prospects of MXenes for wireless communication and EM attenuation.

## Antenna Mechanism and EM Attenuation Mechanism

### Antenna Mechanism

Actually, the wavelength of substrate-based antennas is inversely proportional to frequency, and the length of designed antenna is selected according to antenna types and wavelength. *λ* is defined as [25–30]:

1 $$\lambda = \frac{c}{f}$$

Two important indexes to judge the quality of designed antenna are bandwidth and voltage standing wave ratio (VSWR) or return loss of the antenna [31–33]. The VSWR is to quantify the impedance matching at the operating frequency and different thicknesses, which can be calculated by the formula [34, 35]:

2 $${\text{VSWR}} = \frac{{1 + \left| {S_{11} } \right|}}{{1 - \left| {S_{11} } \right|}}$$

where *S*_11_ represents the reflection coefficients. VSWR is the ratio between the peak amplitude and the minimum amplitude of standing wave. Standing wave is caused by any mismatch at the input of antenna, which will cause the input power to be reflected back [36, 37]. VSWR equal to 1 means that there is no standing wave (*S*_11_ =  − infinity), and the antenna is an ideal match.

The research on 2D materials as patch antennas is the most extensive. Different substrates have been proposed for flexible patch antennas, such as rubber, polyethylene, cellulose nanopaper, and others, to achieve improved efficiency. The patch antenna, using natural rubber as substrate, plays a significant role in wireless communication as the mechanical properties of rubber make the antenna flexible. Moreover, an antenna on a polyethylene substrate was designed and fabricated with distinct bending curvature, affording reliable performance within the designed C-band [38].

### Shielding Mechanism

The ability of a shield to against the incoming EM radiation is measured by the EMI SE, which is defined as the ratio of the transmitted and incident powers, generally on a logarithmic scale, as expressed in Eq. (3) [39]:

3 $${\text{SE}}_{{\text{T}}} \left( {{\text{dB}}} \right) = {\text{10log}}\frac{{P_{{\text{T}}} }}{{P_{{\text{I}}} }} = {\text{20log}}\frac{{E_{{\text{T}}} }}{{E_{{\text{I}}} }}$$

where *P*_I_ and *P*_T_ represent incident wave power and transmitted wave power, respectively. *E*_I_ and *E*_T_ represent electric field intensity of incident wave and electric field intensity of transmitted wave power, respectively. According to the theory of Schelkunoffs, the total EMI SE is the sum of reflection (*S*_R_), absorption (SE_A_), and multiple reflections (SE_M_), as shown in Eq. (4) [40]:

4 $${\text{SE}}_\text{T} = {\text{SE}}_\text{R} + {\text{SE}}_\text{A} + {\text{SE}}{}_\text{M}$$

To quantify the value of SE_R_ and SE_A_, the concept of the absorption (*A*), reflection (*R*) and transmission (*T*) coefficient is proposed. The relation among them can be obtained as follows:

5 $${\text{SE}}_\text{R} = {\text{10log}}\left( {\frac{{1}}{{{1} - R}}} \right)$$

6 $${\text{SE}}_\text{A} = {\text{10log}}\left( {\frac{{{1} - R}}{T}} \right)$$

The value of *A*, *R,* and *T* can be calculated from the measured scattering parameters (*S*_mn_). *S*_mn_ represents that how energy is scattered from a shield. “*m*” indicates the port receiving the radiation energy, and “*n*” indicates the port that is transmitting the incident energy. Accordingly, *A*, *R* and *T* can be calculated via the relationships:

7 $$A + R + T = 1$$

8 $$R = \left| {S_{{{11}}} } \right|^{{2}} = \left| {S_{{{22}}} } \right|^{{2}}$$

9 $$T = \left| {S_{{{21}}} } \right|^{{2}} = \left| {S_{{{12}}} } \right|^{{2}}$$

**Fig. 2:**
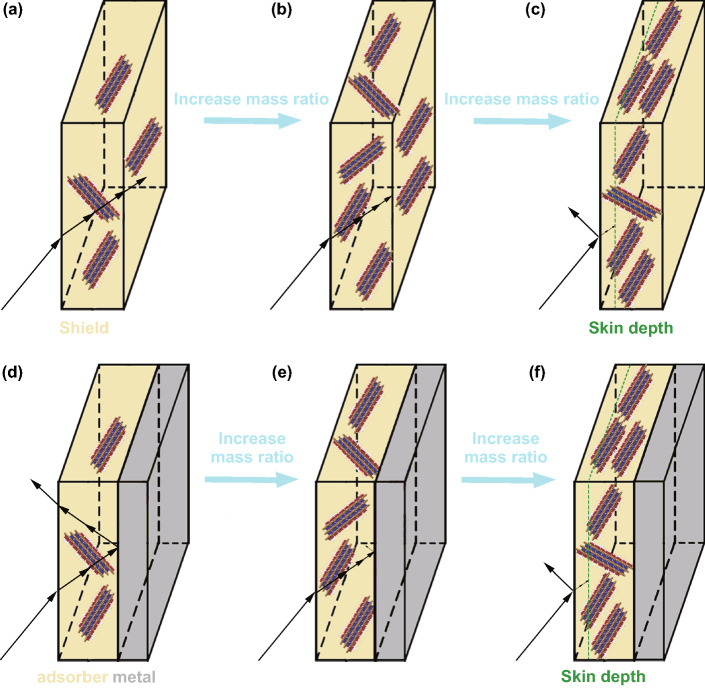
Situations in which the EMI shield responds to the EM wave: **a** allowing all waves to enter with large transmission, **b** allowing all waves to enter with no transmission, and **c** strong secondary reflection. The situations in which the absorber responds to the EM wave: **d** good impedance matching with weak attenuation, **e** an ideal absorber with good impedance matching and strong attenuation, and **f** poor impedance matching with strong attenuation

Both reflection and absorption provide great contributions for excellent EMI shielding. However, in consideration of green shielding, less reflection is better. The ideal EMI shielding involves strong absorption with no reflection and transmission, as shown in Fig. 2b. Multiple reflection also plays an important role in EM wave attenuation. Multiple reflections between the front and back of the shield contribute a lower EMI SE. However, when the thickness of the shield close to or larger than the skin depth ($\delta = \left( {\sqrt {\pi f\mu_{{\text{o}}} \mu_{{\text{r}}} \sigma } } \right)^{ - 1}$, where µ_o_ = 4 × 10^−7^H m^−1^, *µ*_r_ is permeability of an absorber, and *σ* is electrical conductivity) or when the SE_T_ > 10 dB, the effect of multiple reflection should be neglected.

Specific shielding effectiveness (SSE) is derived to compare the effectiveness of shielding materials taking into account the density. Lightweight materials afford high SSE. SSE can be calculated as follows:

10 $${\text{SEE}} = {\text{EMI}}\,{\text{ SE/density}} = {\text{dB}}\,{\text{ cm}}^{{3}} \,{\text{g}}^{{ - {1}}}$$

To account for the thickness contribution, the following equation is used to evaluate the absolute effectiveness (SSE_t_) of a material in relative terms

11 $${\text{SSE}}_\text{t} = {\text{SSE/}}t = {\text{dB }}\,{\text{cm}}^{{2}} \,{\text{g}}^{{ - {1}}}$$

### Absorption Mechanism

The responses of an EM wave absorption to an incident EM wave are determined by the absorber’s permittivity and permeability. The EM wave absorption capacity can be expressed by the following equations [41]:

12 $${\text{RL}} = {\text{20 log}}\left| {\frac{{Z_{{{\text{in}}}} - {1}}}{{Z_{{{\text{in}}}} + 1}}} \right|$$

13 $$Z_{{{\text{in}}}} = \sqrt {\frac{{\mu_{{\text{r}}} }}{{\varepsilon_{{\text{r}}} }}} \tanh \left[ {j\left( {\frac{2f\pi d}{c}} \right)\sqrt {\mu_{{\text{r}}} \varepsilon_{{\text{r}}} } } \right]$$

where *Z*_in_ is the normalized input impedance, *ε*_r_ and *µ*_r_ are the relative complex permittivity and permeability of an absorber and *d* is the thickness of the absorber.

Excellent EM wave absorption performance of an absorber depends not only on efficient EM wave attenuation but also on impedance matching. As shown in Fig. 2e, the absorber exhibits ideal impedance matching, which means that all EM waves are allowed to penetrate the absorber. And, its high-efficiency EM attenuation is usually derived from dielectric loss and magnetic loss, as well as multi-scattering or multi-reflection.

Generally, the dissipation pathway within the absorber is described as dielectric loss. The dielectric loss can be thought as a sort of friction to the displacement of the subatomic particles, then the passing EM wave attenuates, presenting as the subsequent generation of heat. Dielectric loss is constructed by conduction loss and polarization loss; the relation among them can be expressed by the following formula:

14 $$\varepsilon^{^{\prime\prime}} = \varepsilon_\text{c}^{{^{^{\prime\prime}} }} + \varepsilon_\text{p}^{{^{^{\prime\prime}} }}$$

where *ε"*_c_ and *ε"*_p_ represent conduction loss and polarization loss, respectively.

Conduction loss plays an important role in the materials with high conductivity, such as, MXene, graphene and other carbon materials. Actually, the conduction loss is the energy loss of EM wave caused by electron transition. Up to now, Cao’s group has done a lot of work to clarify the role of conduction loss. For example, the electron-hopping model (EHM) was established to explain the mechanism of conduction loss in the carbon fibers (CFs) and multi-walled carbon nanotubes (MWCNTs) [42]; a model of aggregation-induced charge transport (AICT) was proposed to illustrate electron transport in whole MWCNTs/SiO_2_ composites [43]; electron transition theory was used to explain the loss behavior of Ti_3_C_2_T_*x*_ MXene [44]. Now, the role of conduction loss caused by electron transition has been widely recognized in the design of EM wave-absorbing materials.

Polarization loss is generated by the behavior of diploes. Diploes are generated in the site of functional groups, defects, and interfaces. Under a high-frequency alternating electric field, when rotation of dipoles cannot follow the change of electric field, dipole orientation polarization loss occurs, which is another key role of dielectric loss. Cao’s group made important contributions to the resources, characterization techniques, and semiquantitative methods of polarization relaxation. In 2008, they proposed a capacitor-like model and an equivalent circuit model to explain the EM wave response of CdS-Fe_2_O_3_ heterostructures [45]. In 2012, the perfect polarization relaxation was established in Fe_3_O_4_-MWCNTs and PANI-Fe_3_O_4_-MWCNTs [46]. Later, the capacitor-like model is applied to visualize the interfacial polarization in MWCNTs, graphene, MXene, or their nanohybrids, etc. After that, a semiquantitative research strategy of multiple polarization, that is the separation of the contribution of electron transport and dipole polarization, was established by them to accurately analyze the source of polarization [47]. Recently, they proposed a new concept of polarization genes and made a semiquantitative characterization and definition [48]. In conclusion, on the premise of meeting the impedance matching, effectively rising the conduction loss and polarization loss will greatly improve the EM wave absorbing performance of absorber.

Magnetic loss is caused by eddy currents, natural resonance, and exchange resonance. The eddy current is inevitable. If magnetic loss only originates from eddy current loss, the value of *µ"*(*µ'*)^−2^*f*^−1^ is constant when frequency changes. Natural resonance usually occurs at low frequency (2–10 GHz), while exchange resonance occurs at high frequencies (> 10 GHz).

## MXene-Based Materials for Antenna

Research on MXenes for wireless communication applications is still in the exploratory stage. Gogotsi’s group has undertaken pioneering research in this field. According to the different usage frequency, MXene antennas can be divided into low-frequency antennas (< 2.4 GHz) and high-frequency antennas (> 5.6 GHz).

### Low-Frequency Antenna

Gogotsi’s group firstly designed and investigated the Ti_3_C_2_T_*x*_ MXene dipole antenna at 2.4 GHz [49]. The translucent MXene antenna with a thickness of ~ 100 nm had a reflection coefficient of less than − 10 dB. By increasing the antenna thickness to 8 µm, the reflection coefficient reached − 65 dB. The VSWR was less than 2 for MXene antennas with various thicknesses despite the fact that the surface resistance increased significantly with thicknesses below 100 nm. The radiation pattern of an 8-mm-thick dipole antenna presented typical dipole radiative behavior. Moreover, the MXene antenna afforded a maximum gain of 2.11 dB at the thickness of 8 µm, which converges with the maximum gain of an ideal half-wavelength dipole antenna (2.15 dB).

**Fig. 3:**
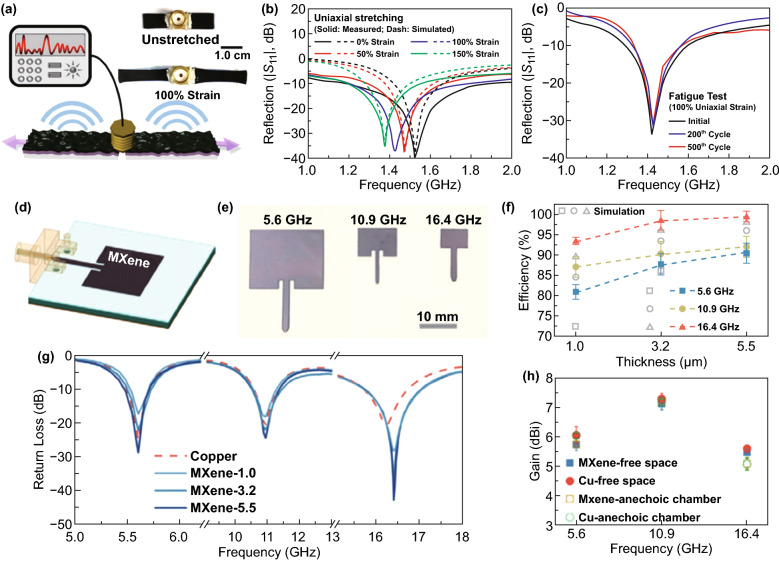
**a** Schematic illustration and digital photographs (right) of an S-MXene dipole antenna at different stretching states. **b** Measured and simulated reflection |*S*_11_| and resonant frequencies of an S-MXene antenna under different uniaxial strains. **c** Performance of stretchable S-MXene antenna under 200- and 500-cycle fatigue tests with uniaxial strain of up to 100%. Reproduced with permission from Ref. [50]. Copyright 2019, WILEY–VCH. **d** Schematic of a MXene microstrip patch antenna on dielectric substrate with SMA connector. **e** Digital image of as-fabricated MXene patch antennas at three target frequencies (5.6, 10.9, and 16.4 GHz), showing the geometry and size. **f** Return loss of MXene patch antennas of different thicknesses (1.0, 3.2, and 5.5 µm) at different target frequencies; copper patch antennas with the same geometry and dimensions were used as references. **g** Measured and simulated radiation efficiency of MXene patch antennas of different thicknesses at different frequencies. **h** The maximum gain of 5.5-µm-thick MXene antenna and a 35-µm-thick copper antenna at different frequencies measured in free space and an anechoic chamber, respectively. Reproduced with permission from Ref. [51]. Copyright 2020, WILEY–VCH

Li et al. prepared a stretchable Ti_3_C_2_T_*x*_ nanosheets (MXene) and single-walled carbon nanotubes (SWNTs) S-MXene dipole antenna (Fig. 3a) [50]. The resonant frequencies of an S-MXene antenna were linearly dependent on the applied strains (Fig. 3b). Moreover, the S-MXene antenna afforded nearly the same reflection |*S*_11_| (~ − 33 dB) at the same resonant frequency (1.425 GHz) during the fatigue test up to 100% uniaxial strains for 500 cycles (Fig. 3c).

### High-Frequency Antenna

Gogotsi’s group reported on micrometer-thin and flexible MXene microstrip patch antennas with target frequencies of 5.6, 10.9, and 16.4 GHz, produced by a simple spray-coating fabrication method (Fig. 3d, e) [51]. The return loss values of MXene antennas with a thickness of 5.5 µm achieved − 29, − 25, and − 48 dB at 5.6, 10.9, and 16.4 GHz (Fig. 3f), respectively, which demonstrated that MXene patches were capable of delivering RF power efficiently to the radiator. The radiation efficiency increased with the thickness of MXene patches (Fig. 3g), which was due to the decreasing conductor loss. Moreover, the outstanding performance of MXene patch antennas was comparable to their copper counterparts (Fig. 3h).

## MXene-Based Materials for EMI Shielding

### Pure MXene Matrix

#### Non-annealing

Liu et al. prepared multilayer Ti_3_C_2_T_*x*_ by etching Ti_3_AlC_2_ with 40% HF at room temperature (RT) for 24 h [52]. The multilayer Ti_3_C_2_T_*x*_/wax with the 60 wt% Ti_3_C_2_T_*x*_ content displayed outstanding EMI shielding performance of 39.1 dB at the thickness of 2 mm. Hu et al. fabricated multilayer Ti_3_C_2_T_*x*_ by etching Ti_3_AlC_2_ with 40% HF solution at 50 °C for only 0.5 h [53]. The multilayer Ti_3_C_2_T_*x*_ prepared in such a short time also exhibited excellent EMI shielding performance. For example, the Ti_3_C_2_T_*x*_/wax with 70 wt% Ti_3_C_2_T_*x*_ showed EMI shielding performance of 34 dB at 18 GHz.

**Fig. 4:**
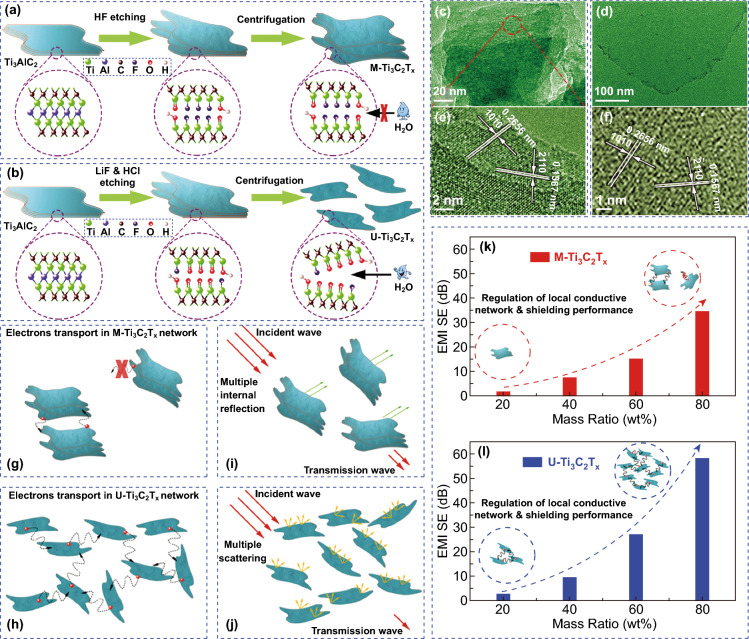
Synthetic illustration of **a** multilayer Ti_3_C_2_T_x_ and **b** Ti_3_C_2_T_x_ nanosheet. The high-magnification TEM images of **c** multilayer Ti_3_C_2_T_x_ **d** Ti_3_C_2_T_x_ nanosheet. HRTEM images of **e** multilayer Ti_3_C_2_T_x_ and **f** Ti_3_C_2_T_x_ nanosheet. Local conductive network of **g** multilayer Ti_3_C_2_T_x_ and **h** Ti_3_C_2_T_x_ nanosheet. Microwave propagation model in **i** multilayer Ti_3_C_2_T_x_ and **j** Ti_3_C_2_T_x_ nanosheet. Regulation of local conductive network and shielding performance for **k** multilayer Ti_3_C_2_T_x_ and **l** Ti_3_C_2_T_x_ nanosheet. Reproduced with permission from Ref. [54]. Copyright 2019, The Royal Society of Chemistry

Previous studies focused on the EMI shielding performance of multilayer Ti_3_C_2_T_*x*_. He et al. investigated the difference between Ti_3_C_2_T_*x*_ nanosheet and multilayer Ti_3_C_2_T_*x*_ in EMI shielding performance [54]. Different etchants led to different centrifugal results (Fig. 4a, b) and different morphologies (Fig. 4c–f). The Ti_3_C_2_T_*x*_ nanosheet showed a much better EMI shielding performance compared with the multilayer Ti_3_C_2_T_*x*_ owing to the formation of local conductive networks (Fig. 4g–l). The Ti_3_C_2_T_*x*_-wax matrix with 80 wt% Ti_3_C_2_T_*x*_ loading showed EMI shielding performance of 58.1 dB at the thickness of only 1 mm.

Li et al. prepared the multilayer Ti_2_CT_*x*_ MXene by HCl/LiF etching Ti_2_AlC for 48 h at 40 °C [55]. The multilayer Ti_2_CT_*x*_ presented outstanding EMI shielding performance of 70 dB with a thickness of only 0.8 mm in the X-band, exceeding most of reported graphene-based EMI shielding composites, owing to the multilayered structure and the high electrical conductivity (0.30 S cm^−1^).

The matrix of EMI-absorbing materials is usually made of wave-transparent materials. The imaginary part of the dielectric constant of the matrix is zero, which means there is no EM loss. Paraffin is generally used as the matrix of absorbing materials. There are also other wave-transmitting materials that can be used as the matrix, such as polystyrene (PS) and polyvinylidene fluoride (PVDF). A Ti_3_C_2_T_*x*_ nanosheet@ PS matrix was fabricated by electrostatic assembling of negative Ti_3_C_2_T_*x*_ nanosheet on positive polystyrene microsphere [56]. The Ti_3_C_2_T_*x*_@PS with 1.9 vol% Ti_3_C_2_T_*x*_ loading exhibited an outstanding EMI shielding performance of > 54 dB over the whole X-band. Such excellent EMI shielding performance was attributed to the high conductivity (1081 S m^−1^) of Ti_3_C_2_T_*x*_ and their highly efficient conducting network within PS matrix. Rajavel et al. reported that the multilayer Ti_3_C_2_T_*x*_-PVDF with 22.55 vol% Ti_3_C_2_T_*x*_ displayed remarkable shielding performance of 48.47 ± 3.5 dB with a thickness of 2 mm [57]. Such outstanding shielding performance was attributed to the formation of conducting network along with the assembly of micro-capacitor network.

#### Annealing

The improvement of EMI shielding properties by annealing is also widely studied. Yin group investigated the EMI shielding performance of multilayer Ti_3_C_2_T_*x*_ annealed at 800 °C (Ti_3_C_2_T_*x*_-800) in Ar atmosphere. The total SE of Ti_3_C_2_T_*x*_-800/wax was 32 dB [58]. After that, the Ti_3_C_2_T_*x*_-200 was fabricated by annealing Ti_3_C_2_T_*x*_ at 200 °C for 2 h in Ar + 5% H_2_ atmosphere [59]. The Ti_3_C_2_T_*x*_-200/epoxy displayed enhanced EMI shielding performance compared with Ti_3_C_2_T_*x*_/epoxy. Ji et al. investigated the EMI shielding performance of multilayer Ti_3_C_2_T_*x*_ at different annealing temperatures (800, 950, 1100, 1250, 1400, and 1550 °C for 1 h) in Ar atmosphere [60]. Among them, the multilayer Ti_3_C_2_T_*x*_ annealed at 1100 °C displayed the best EMI shielding performance, showing excellent EMI shielding performance of 76.1 dB.

### MXene Hybrid Matrix

It is common that MXene is doped with one-dimensional nanoparticles. Ti_3_C_2_T_*x*_ nanosheet/Ni chain hybrid was fabricated by a one-step hydrothermal process [61]. The Ti_3_C_2_T_*x*_/Ni hybrid with 50 wt% Ti_3_C_2_T_*x*_ showed EMI SE of 66.4 dB. The excellent EMI shielding performance was due to the synergistic effect of conductive Ti_3_C_2_T_*x*_ and magnetic Ni chains, by which the dielectric properties and EM loss can be easily controlled to obtain good EM wave dissipation ability. Nb_2_CT_*x*_/Nb_2_O_5_-Ag ternary hybrid nanostructures were fabricated by self-reduction and oxidation of Nb_2_CT_*x*_ in the presence of metallic salt (AgNO_3_) [62]. The Nb_2_CT_*x*_/Nb_2_O_5_-Ag hybrid-wax matrix with a thickness of 1 mm showed excellent EMI SE of 68.76 and 72.04 dB in the X- and Ku-band region, respectively. The excellent EMI shielding performance was attributed to the strong electrical conductivity, increased interface polarization, and multiple reflection loss between the ternary interfaces.

MXene is not only doped with one-dimensional nanoparticles, but also doped with two-dimensional materials. Song et al. investigated the EMI shielding performance of honeycomb structural rGO–Ti_3_C_2_T_*x*_ MXene/epoxy nanocomposites [63]. The introduction of rGO–Ti_3_C_2_T_*x*_ can fully play synergistic effects of rGO and MXene, to greatly improve the electrical conductivity and EMI SE of rGO–Ti_3_C_2_T_*x*_ MXene/epoxy nanocomposites. When the cell size of 0.5 mm with 1.2 wt% rGO + 3.3 wt% Ti_3_C_2_T_*x*_ loading, the enhanced σ (387.1 S m^−1^) and EMI SE (55 dB) values were reached, which were, respectively, 2978 and 5 times of rGM/epoxy nanocomposites (σ of 0.13 S m^−1^, EMI SE of 11 dB) at the same loading of directly blending rGO–Ti_3_C_2_T_*x*_ fillers.

The shielding properties of multi-doped MXene have also been studied. Raagulan fabricated the Ti_3_C_2_T_*x*_ MXene-p-aminophenol (PAT)-conductive polymer (CP) by a cost-effective spray coating technique and characterization [64]. The composite showed excellent EMI shielding performance of 45.18 dB and good electric conductivity of 7.813 S cm^−1^.

### Film

The EMI shielding properties of MXene as a film have been widely studied, and there are numerous research results. As a shield, MXene films can be divided into three categories: pristine MXene films, organic-hybrid MXene films, and inorganic-hybrid MXene films. The pristine MXene film refers to the film fabricated from pure MXene nanosheets, organic-hybrid MXene films contain organic substances, and inorganic-hybrid MXene films are MXene films doped with an inorganic substance.

#### Pristine

Koo’s group reported that a 45-µm Ti_3_C_2_T_*x*_ film displayed EMI SE of 92 dB (a 2.5-µm film showed > 50 dB), which is the highest among synthetic materials of comparable thickness produced to date [65]. The outstanding electrical conductivity of Ti_3_C_2_T_*x*_ films and multiple internal reflections led to this excellent performance. After that, they systematically studied the EMI shielding of Ti_3_C_2_T_*x*_ MXene-assembled films over a broad range of film thicknesses, monolayer by monolayer [66]. Theoretical research showed that multiple reflection, the surface reflection, and bulk absorption become significant in the shielding mechanism below skin depth. The 24-layer film of 55 nm thickness showed EMI SE of 20 dB, revealing an extraordinarily large absolute shielding effectiveness (3.89 × 106 dB cm^2^ g^−1^). Meanwhile, they prepared Ti_3_CNT_*x*_ and Ti_3_C_2_T_*x*_ MXene free-standing films of different thicknesses by vacuum-assisted filtration and investigated their EMI shielding performance under different annealing temperatures [67]. It is found that Ti_3_CNT_*x*_ film provided a higher EMI SE compared with more conductive Ti_3_C_2_T_*x*_ or metal foils of the same thickness. This excellent EMI shielding performance of Ti_3_CNT_*x*_ was achieved by thermal annealing, owing to an anomalously high absorption of EM waves in its layered, metamaterial-like structure.

Han et al. systematically studied the shielding properties of 16 different MXene films [68]. All MXene films with micrometer thick displayed excellent EMI shielding performance (> 20 dB). Among them, Ti_3_C_2_T_*x*_ film displayed the best EMI shielding performance. For example, Ti_3_C_2_T_*x*_ film with a thickness of only ~ 40 nm showed the EMI shielding performance of 21 dB.

#### Organic-Hybrid

The organic substances for hybridization can be divided into three categories: aramid nanofibers (ANFs), cellulose nanofibers (CNFs), and others.

##### ANF

Xie et al. prepared Ti_3_C_2_T_*x*_ nanosheet/ANF composite film by vacuum-assisted filtration approach [69]. The Ti_3_C_2_T_*x*_/ANF composite film with different Ti_3_C_2_T_*x*_ addition exhibited outstanding EMI shielding performance, which was accomplished beyond the commercial standard for EMI shielding materials. The 80%-Ti_3_C_2_T_*x*_/ANF composite film with an ultra-thin thickness ~ 17 µm possessed an EMI SE of ~ 28 dB in 8.2–12.4 GHz and electrical conductivity of 173.36 S cm^−1^. Weng et al. further confirmed this conclusion [70]. Lei et al. found that the Ti_3_C_2_T_*x*_/ANF composite film with a loading of 40 wt% Ti_3_C_2_T_*x*_ showed high electrical conductivity of 3661.8 S m^−1^ [71], and excellent EMI shielding performance of 24.5 dB, and SEE_t_ of 8814.5 dB cm^2^ g^−1^ at the thickness of 14 µm. Wei et al. reported that the ANF/Ti_3_C_2_T_*x*_ film with 90 wt% displayed the EMI SE of 34.71 dB at the thickness of 11 µm and SEE_t_ of 21,971.37 dB cm^2^ g^−1^, which would be no recession after 1000 times bending [72].

After that, ternary mixed ANF-MXene film was reported [73]. A double-layered and homogeneously blended ANF-Ti_3_C_2_T_*x*_ MXene/silver nanowire (ANF-MXene/AgNW) nanocomposite film was fabricated via the facile two-step vacuum-assisted filtration followed by hot-pressing approach, respectively. Compared with the homogeneously blended ones, the double-layered nanocomposite papers possessed greater advantages in EMI shielding performances, which was due to the massive ohmic losses, multiple internal reflections and polarization relaxation of localized defects, and abundant terminal groups.

##### CNF

Cao et al. fabricated an ultrathin and highly flexible delaminated Ti_3_C_2_T_*x*_ (d-Ti_3_C_2_T_*x*_ MXene)/CNF composite film through a vacuum-filtration-induced self-assembly process [74]. The *d*-Ti_3_C_2_T_*x*_/CNF film with 90 wt% *d*-Ti_3_C_2_T_*x*_ content showed high electrical conductivity (739.4 S m^−1^) and outstanding EMI shielding performance (SEE_t_ = 2647 dB cm^2^ g^−1^). Zhou et al. found that the CNF/Ti_3_C_2_T_*x*_ film showed EMI SE of ~ 40 dB and high SEE_t_ up to 7029 dB cm^2^ g^−1^ with a thickness of only 0.035 mm [75]. Moreover, the EMI shielding properties could withstand the folding test more than 1000 times without obvious reduction. Cui et al. reported that a Ti_3_C_2_T_*x*_/CNF film exhibited EMI SE of 42.7 dB with a thickness of 15 µm, owing to the high electrical conductivity (46.3 S cm^−1^) [76].

Zhou et al. fabricated a CNF/Ti_3_C_2_T_*x*_/g-C_3_N_4_ film with Ti_3_C_2_T_*x*_/g-C_3_N_4_ mass ratio of 5:1 presented outstanding performance with EMI SE of 42.99 dB in X-band at the thickness of 28.20 µm [77]. Moreover, both the electrical conductivity and the EMI SE of the film remained nearly unchanged after bending at 135° for 10,000 cycles.

Xin et al. investigated the EMI shielding performance of a Ti_3_C_2_T_*x*_/CNF/silver composite film [78]. The Ti_3_C_2_T_*x*_/CNF/silver composite film exhibited excellent EMI shielding performance (50.7 dB) and good electrical conductivity (588.2 S m^−1^), attributed to Ti_3_C_2_T_*x*_ MXene, self-reduction of silver nanoparticles, and the brick-like structure.

An ultrathin and flexible carbon nanotubes/Ti_3_C_2_T_*x*_/CNF composite film was fabricated via a facile alternating vacuum-assisted filtration process [79]. The film showed a high electrical conductivity of 2506.6 S m^−1^ and EMI SE of 38.4 dB. This result was attributed to the sandwich structure in improving EMI SE, and the gradient structure on regulating the contributions from refection and absorption.

##### Others

**Fig. 5:**
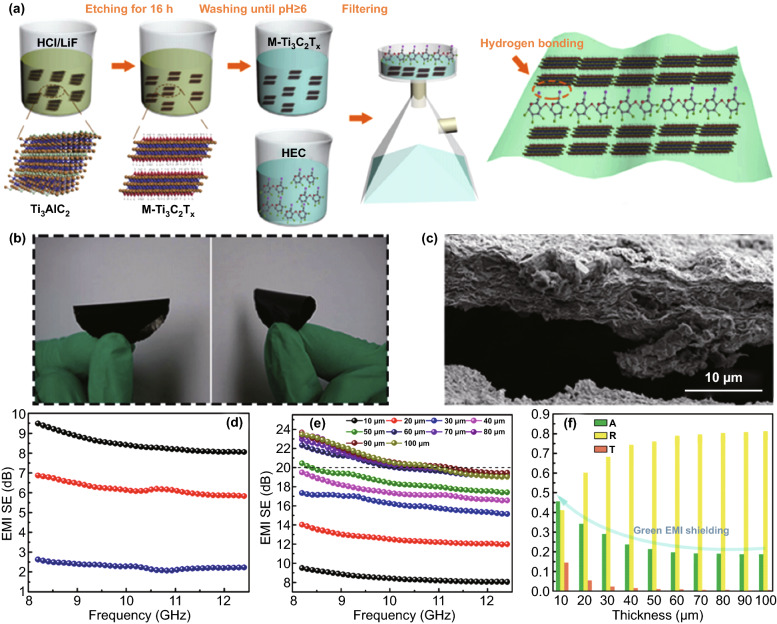
**a** Schematic illustration of fabricating M-Ti_3_C_2_T_x_/HEC composite film. **b** Digital image of M-Ti_3_C_2_T_x_/HEC composite film. **c** Cross-sectional SME images of M-Ti_3_C_2_T_x_/HEC composite film. **d** EMI SE_T_, SE_A_ and SE_R_ of M-Ti_3_C_2_T_x_/HEC composite film at 10 µ. **e** EMI SE of M-Ti_3_C_2_T_x_/HEC composite film at different stacking thicknesses. **f** The average A, R and T of M-Ti_3_C_2_T_x_/HEC composite film at different stacking thicknesses. Reproduced with permission from Ref. [80]. Copyright 2019, Elsevier

The flexible green multilayered Ti_3_C_2_T_*x*_/hydroxyethyl cellulose (M-Ti_3_C_2_T_*x*_/HEC) composite film was prepared via the filtration-assisted self-assembly method (Fig. 5a–c) [80]. The effect of multilayer stacking on the EMI shielding performance was investigated. The EMI SE of the film exceeded 20 dB under the stacking thickness reaching 100 mm (Fig. 5d, e). Notably, the film presented the trend of absorption-dominate green EMI shielding with the decrease of the stacking thickness (Fig. 5f).

Gogotsi’s group investigated the EMI shielding performance of a polyethylene terephthalate (PET)/Ti_3_C_2_T_*x*_ nanosheet film [81]. The PET/Ti_3_C_2_T_*x*_ film exhibited electrical conductivity values of 1080 ± 175 S cm^−1^, which profoundly exceeded electrical conductivity values of other 2D materials including graphene (250 S cm^−1^) and reduced graphene oxide (340 S cm^−1^). Such excellent EMI shielding performance was ascribed to the high electrical conductivity and layered structure.

A free-standing, ultrathin, and flexible Ti_3_C_2_T_*x*_/poly (3,4-ethylenedioxythiophene)-poly (styrene sulfonate) (PEDOT: PSS) film was prepared by a vacuum-assisted filtration process [82]. The composite film with 11.1 µm prepared a high EMI SE value of 42.1 dB. Meanwhile, the hybrid film exhibited a superior conductivity of 340.5 S cm^−1^ and an excellent specific EMI shielding efficiency of 19,497.8 dB cm^2^ g^−1^. This result was due to the lamellar structure of the films and multiple interface reflection and polarization.

Luo et al. fabricated a flexible Ti_3_C_2_T_*x*_ nanosheet/natural rubber (NR) nanocomposite film via vacuum-assisted filtration approach [83]. In the film, Ti_3_C_2_T_*x*_ nanosheets selectively distributed at the interfaces of the NR particles, forming an interconnected network for efficient electron transport, which leads to excellent EMI shielding performance. The Ti_3_C_2_T_*x*_ nanosheet/NR film with 6.71 vol% of Ti_3_C_2_T_*x*_ nanosheet showed an outstanding electrical conductivity of 1400 S m^−1^ and a superb EMI shielding performance of 53.6 dB.

Wang et al. prepared a flexible and ultrathin poly (vinylidene fluoride) (PVDF)/Ti_3_C_2_T_*x*_/Ni chain composite film by physical mixing [84]. The PVDF/Ti_3_C_2_T_*x*_/Ni chain composite film with only 0.10 mm thickness showed EMI shielding performance of 19.3 dB, which increased to 34.4 dB at 0.36 mm thickness. The outstanding EMI shielding performance was attributed to the excellent electrical conductivity (892 S m^−1^).

An ultrathin Ti_3_C_2_T_*x*_/calcium alginate (CA) aerogel film was fabricated via divalent metal ion-induced crosslinking, vacuum-assisted filtration, and freeze-drying. The Ti_3_C_2_T_*x*_/CA aerogel film with a thickness of 26 µm presented excellent EMI SE (54.3 dB), owing to its sponge-like structure, which facilitated the dissipation of incident EM waves through multi-reflection and scattering in the Ti_3_C_2_T_*x*_/CA aerogel film [85].

Liu et al. fabricated a polyurethane/Ti_3_C_2_T_*x*_ MXene film vacuum-assisted filtration [86], thanks to the bioinspired material design and the careful choice of polyurethane as a polymer matrix. The polyurethane/Ti_3_C_2_T_*x*_ with nacre-like structure showed superior electric conductivity of ~ 2897.4 S cm^−1^ and SEE_t_ of 33,771.92 dB cm^2^ g^−1^ with ultra-small thickness (< 10 µm).

A poly vinyl alcohol/Ti_3_C_2_T_*x*_ (PVA/Ti_3_C_2_T_*x*_) film with alternating multilayered structure was prepared by multilayered casting [87]. When the amount of Ti_3_C_2_T_*x*_ was 19.5 wt%, PVA/Ti_3_C_2_T_*x*_ multilayered film with a thickness of 27 µm displayed electrical conductivity of 716 S m^−1^, EMI SE of 44.4 dB, and the SEE_t_ of 9343 dB cm^2^ g^−1^. This excellent performance was due to the improved multiple interfacial reflection and improved absorption in the MXene layer.

Liu et al. prepared a chitosan (CS)/Ti_3_C_2_T_*x*_ film by vacuum-assisted filtration [88]. The CS/Ti_3_C_2_T_*x*_ film with the Ti_3_C_2_T_*x*_ content of 75 wt% displayed high EMI shielding performance of ~ 34.7 dB at the thickness of 13 µm, which was attributed to the outstanding electrical conductivity (~ 1402 ± 70 S m^−1^) and multiple internal reflection.

A Ca ion cross-linked sodium alginate (SA)-montmorillonite (MMT)/Ti_3_C_2_T_*x*_ MXene (CSA-M-T) film was fabricated by a step-by-step vacuum-assisted filtration process [89]. Compared with the pure Ti_3_C_2_T_*x*_ layer, such kind of sandwich film can effectively maintain the EMI shielding performance (50.01 dB).

#### Inorganic Hybrid

##### Carbon-Based

A assemble Ti_3_C_2_T_*x*_ MXene-carbon nanotube (CNT) composite film was fabricated by spin spray layer-by-layer (LbL) [90]. The absolute effectiveness of the film was up to 58,187 dB cm^2^ g^−1^, which was due to the both the excellent electrical conductivity (130 S cm^−1^) and the enhanced absorption with the LbL architecture of the films.

Xiang et al. prepared a lightweight and ultrathin TiO_2_-Ti_3_C_2_T_*x*_/graphene film with the range of 5.25–9.17 µm of thickness by vacuum filtration and pyrolysis [91]. The film displayed surface resistance of 7.5 Ω sq^−1^ and EMI SE of 27 dB. Meanwhile, the value of SEE_t_ of the film reached 30,291.43 dB cm^2^ g^−1^.

A stretchable Ti_3_C_2_T_*x*_ nanosheet/single-walled carbon nanotube (SWNT) film was fabricated by Li et al. by pre-buckling of SWNT and Ti_3_C_2_T_*x*_ nanosheet coatings on latex substrates [50]. A single layer of stretchable Ti_3_C_2_T_*x*_/SWNT film demonstrated a strain-invariant EMI shielding performance of ≈ 30 dB up to 800% areal strain.

Zhang’s group investigated the EMI shielding performance of Ti_3_C_2_T_*x*_ MXene-GO film [92]. Thanks to the high electronic conductivity, the Ti_3_C_2_T_*x*_-GO film with a small film thickness of 7 µm displayed the EMI SE of 50.2 dB. Compared with other shielding materials, the MXene-GO films were obviously superior in combining excellent EMI shielding performance and good tensile strength.

A Ti_3_C_2_T_*x*_-bonded carbon black (CB) film with a porous structure was fabricated by a vacuum-assisted filtration method [93]. With the incorporation of 70 mg of CB, the Ti_3_C_2_T_*x*_-bonded CB film showed EMI shielding performance of 60 dB with a SE_A_ of 15 dB and SE_R_ of 45 dB. Moreover, the SEE_t_ reached 8718 dB cm^2^ g^−1^. Research showed that the porous structure could improve the absorption, resulting from enhanced scattering and reflection.

##### Others

A aluminum ion-reinforced Ti_3_C_2_T_*x*_ MXene (Al-Ti_3_C_2_T_*x*_) film was fabricated by Zhang group via vacuum filtration method [94]. The Al-Ti_3_C_2_T_*x*_ film displayed a high conductivity of 265,600 S m^−1^. The strong and highly conductive MXene film with a small thickness of 39 mm showed EMI shielding performances of over 80 dB in the X-band.

Ning et al. investigated the EMI shielding performance of a Mn ion-intercalated Ti_3_C_2_T_*x*_ (MIT) film [95]. The MIT film showed an average electronic conductivity of 4268 S m^−1^, which was two times than that of pure Ti_3_C_2_T_*x*_ film (1894 S m^−1^). The MIT film with a thickness of 2.5 µm showed enhanced performance of 44.3 dB compared with pure Ti_3_C_2_T_*x*_ film (24.1 dB), owing to the additive internal absorption.

A sliver nanowire (AgNW)/Ti_3_C_2_T_*x*_ film was fabricated by a pressured-extrusion film-forming process [96]. The MXene/AgNW composite film with a low loading of nanocellulose (0.167 wt%) showed high electrical conductivity of ~ 30,000 S m^−1^, and remarkable SEE_t_ of 16,724 dB cm^2^ g^−1^.

A Ti_3_C_2_T_*x*_/montmorillonite (MMT) film was fabricated by a simple vacuum-assisted filtration technique [97]. The EMI shielding performance of Ti_3_C_2_T_*x*_/MMT film with different concentration ratios was investigated. The composite film with 10 wt% MMT showed high electrical conductivity (4420 S m^−1^), EMI SE of 65 dB in the entire X-band and SEE_t_ of over 10,000 dB cm^2^ g^−1^ at a thickness of only 25 µm.

### Foam

#### Pristine

**Fig. 6:**
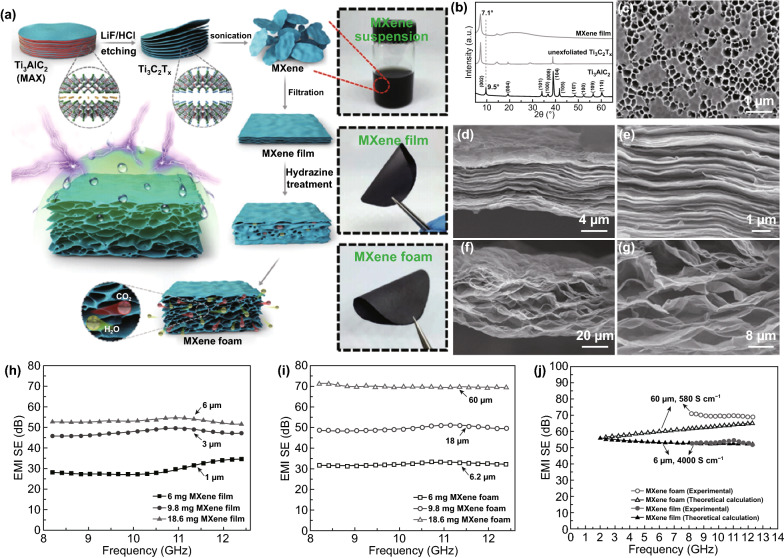
**a** Schematic illustration of the fabrication of the hydrophobic and flexible MXene foam. **b** XRD patterns of Ti_3_AlC_2_, unexfoliated Ti_3_C_2_T_x_, and the MXene film. **c** SEM image of the Ti_3_C_2_T_x_ MXene sheets on an AAO filter. Cross-sectional SEM images of the MXene film **d**, **e** and the MXene foam **f**, **g**. EMI SE of the **h** MXene films and **i** MXene foams with different thicknesses (µm). **j** Experimental and theoretical EMI SE of the MXene foam and bulk MXene film. Reproduced with permission from Ref. [98]. Copyright 2017, WILEY–VCH

The inceptive study of the EMI shielding performance of pure MXene foam was reported by Yu’s group [98]. They used an efficient and facile method to prepare free-standing, flexible, and hydrophobic Ti_3_C_2_T_*x*_ MXene foam (Fig. 6a–g). In striking contrast to well-known hydrophilic MXene materials, the Ti_3_C_2_T_*x*_ foams surprisingly had hydrophobic surfaces, with outstanding water resistance and durability. Thanks to the highly efficient wave attenuation in the favorable porous structure, the lightweight Ti_3_C_2_T_*x*_ foam showed enhanced EMI shielding performance of 70 dB compared with its unfoamed film counterpart (53 dB) (Fig. 6h–j).

#### Hybrid

A porous 3D Ti_3_C_2_T_*x*_ MXene/C hybrid foam (MCF) was prepared by sol–gel followed by thermal reaction [99]. And, the MCF/epoxy was obtained via vacuum-assisted impregnation followed by curing process. The MCF/epoxy with 4.25 wt% MCF displayed the EMI shielding performance of 46 dB and electrical conductivity of 184 S/m, which was 4.8 and 3.1 × 10^4^ times higher than that of MCF-0/epoxy nanocomposites (without Ti_3_C_2_T_*x*_ MXene), respectively.

A porous few-layered Ti_2_CT_*x*_ (f-Ti_2_CT_*x*_) MXene/poly (vinyl alcohol) (PVA) composite foam was fabricated by a facile freeze-drying method [100]. The f-Ti_2_CT_*x*_/PVA foam with a content of only 0.15 vol% afforded a SEE_t_ of 5136 dB cm^2^ g^−1^. Such excellent EMI shielding performance was attributed to the multi-porous structure, internal reflection, and polarization effect.

A lightweight Ti_3_C_2_T_*x*_ MXene/graphene (Ti_3_C_2_T_*x*_-GO) hybrid foam was fabricated by freeze-drying and reduction heat treatment [101]. Thanks to the improved foam electrical conductivity and highly efficient wave attenuation in interconnected porous structures, the Ti_3_C_2_T_*x*_-GO hybrid foam showed excellent EMI shielding performance of 50.7 dB and specific EMI shielding effectiveness of 6217 dB cm^3^ g^−1^, which was much higher than that most of the EMI shielding materials.

Silver nanowires (AgNWs)/Ti_3_C_2_T_*x*_ foam was fabricated by integrating AgNWs as the skeleton and Ti_3_C_2_T_*x*_ as the covering decoration for foaming structure [102]. The AgNWs/Ti_3_C_2_T_*x*_ foam showed EMI shielding performance of 41.3 dB at the thickness of 1.2 µm in the X-band. The freespace created during foaming helped to obtain EM wave scattering within the skin depth.

A polydimethylsiloxane (PDMS)-coated Ti_3_C_2_T_*x*_ MXene foam was fabricated by using the Ti_3_C_2_T_*x*_ assisted with sodium alginate (SA) as template followed by coating a thin layer of PDMS [103]. The Ti_3_C_2_T_*x*_/SA/PDMS foam with 95 wt% Ti_3_C_2_T_*x*_ exhibited an excellent conductivity of 2211 s m^−1^ and EMI shielding performance of 70.5 dB. Moreover, the foam with 74 wt% Ti_3_C_2_T_*x*_ displayed EMI SE of 48.2 dB after 500 compression–release cycles.

### Aerogel

#### Pristine

**Fig. 7:**
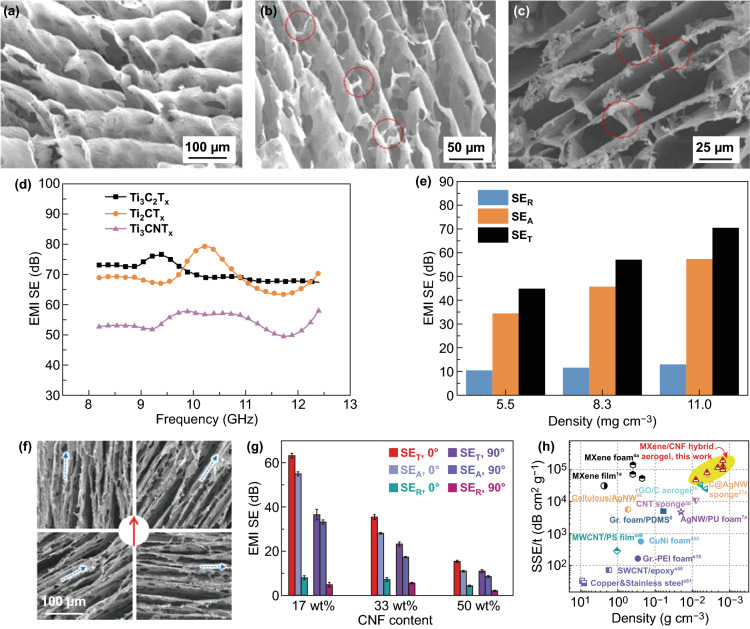
SEM images of Ti_3_C_2_T_x_ aerogels with the densities of **a** 5.5, **b** 8.3, and **c** 11.0 mg cm^−3^, respectively. Interlayer synapses and bridges are circled in red. **d** Frequency-dependent EMI SE of different MXene aerogels at a density of 11.0 mg cm^−3^ and a compression thickness of 1 mm. **e** The average EMI SE of Ti_3_C_2_T_x_ aerogels with different densities. Reproduced with permission from Ref. [104]. Copyright 2019, WILEY–VCH. **f** SEM images of the MXene/CNF hybrid aerogels with various angles between the cell walls’ oriented direction and electric field direction of incident EM waves. **g** EMI shielding performance at a fixed frequency of 10 GHz for the MXene/CNF hybrid aerogels (4 mg cm^−3^) with various CNF contents at cell wall-electric field angles of 0° and 90°, respectively. **h** Comparison of the MXene/CNF hybrid aerogels’ shielding performance with other materials. Reproduced with permission from Ref. [106]. Copyright 2020, WILEY–VCH

Han et al. fabricated three types of porous MXene aerogels (Ti_3_C_2_T_*x*_, Ti_2_CT_*x*_, and Ti_3_CNT_*x*_) via a bidirectional freeze-casting technique (Fig. 7a–c) [104]. The EMI SE of Ti_3_C_2_T_*x*_, Ti_2_CT_*x*_, and Ti_3_CNT_*x*_ aerogels reached 70.5, 69.2, and 54.1 dB at the thickness of 1 mm (Fig. 7d, e), respectively. Especially, the SEE_t_ of Ti_2_CT_*x*_ aerogel with a density of 5.5 mg cm^−3^ and a thickness of 1 mm reached 8818.2 dB cm^2^ g^−1^, which was several times higher than that of other materials. Meanwhile, Bian et al. prepared an ultralight Ti_3_C_2_T_*x*_ aerogel by the freeze-drying method [105]. The Ti_3_C_2_T_*x*_ aerogel with density of 6.26 mg cm^−1^ exhibited electrical conductivity of 22 S cm^−1^ and SEE_t_ of 9904 dB cm^3^ g^−1^. The excellent EMI shielding performance was attributed to the high electrical conductivity and porous structures.

#### Hybrid

A cellulose nanofibril (CNF)/Ti_3_C_2_T_*x*_ aerogel was fabricated via an ice-templated freeze-casting approach (Fig. 7f) [106]. The Ti_3_C_2_T_*x*_ MXene “bricks” bonded by CNF “mortars” of the nacre-like cell walls induced high electrical conductivity, and interfacial polarization led to excellent EMI shielding performance. The CNF/Ti_3_C_2_T_*x*_ aerogel with ultralow density showed EMI SE up to 74.6 dB, SEE of as 30,660 dB cm^3^ g^−1^, and SEE_t_ achieving 189,400 dB cm^2^ g^−1^ (Fig. 7g, h), exceeding that of other MXene-based or other shielding architectures reported so far.

Zhang et al. fabricated a 3D Ti_3_C_2_T_*x*_ MXnen/reduced graphene (RGO) hybrid aerogel by directional freezing and freeze-drying [107]. The Ti_3_C_2_T_*x*_/RGO hybrid aerogel with aligned cellular microstructure displayed a high electrical conductivity of 1085 S m^−1^ and an excellent EMI shielding performance of 50 dB in the X-band at a low Ti_3_C_2_T_*x*_ content of 0.74 vol%, which was the best results among polymer nanocomposites with similar loading of Ti_3_C_2_T_*x*_.

Koo’s group fabricated a 3D porous Ti_3_C_2_T_*x*_/carbon nanotube (CNT) hybrid aerogel by a bidirectional freezing method [108]. The Ti_3_C_2_T_*x*_/CNT aerogel showed excellent electrical conductivity of 9.43 S cm^−1^ and superior EMI shielding performance of 103.9 dB at 3 mm thickness over the X-band frequency. The excellent EMI shielding performance of the Ti_3_C_2_T_*x*_/CNT hybrid aerogel was attributed to the 3D porous structure with a high-conducting and uniform lamellar structure.

Liang et al. prepared a Ti_3_C_2_T_*x*_ MXene/wood-derived porous carbon (WPC) aerogel via freeze-drying procedure [109]. Such wall-like “mortar-brick” structures profoundly prolong the transmission paths of the EM waves and dissipate the incident EM waves in the form of heat and electric energy, thereby exhibiting the superior EMI shielding performance. The Ti_3_C_2_T_*x*_/WPS aerogel showed EMI SE value of 71.3 dB at density as low as 0.197 g cm^−3^.

### Fabric

MXene fabric usually refers to MXene-coated fabric. The main fabrics used for this purpose are cotton or polymers, although other materials are also utilized.

#### Cotton

**Fig. 8:**
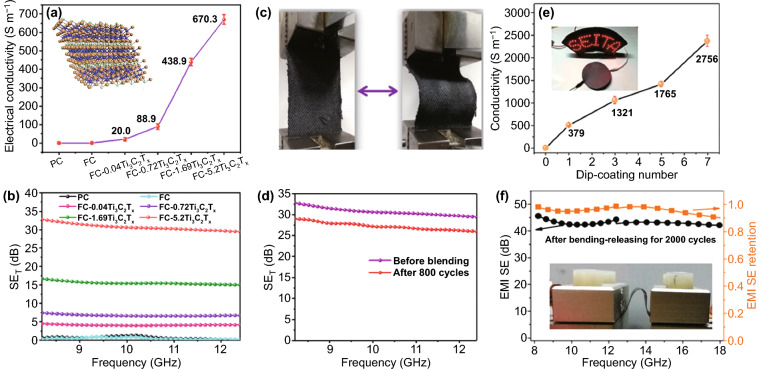
**a** Electrical conductivity and **b** EMI shielding performance of samples in X-band. **c** Process of bending test and **d** EMI shielding performance of FC-5.2Ti_3_C_2_T_x_ before and after 800 cycles bending test. Reproduced with permission from Ref. [112]. Copyright 2020, American Chemical Society. **e** Electrical conductivity of M-filter as a function of dip-coating cycles. **f** EMI SE of the PM-7 after 2000 bending-releasing cycles and the EMI SE retention. Reproduced with permission from Ref. [113]. Copyright 2020, Elsevier

Geng et al. prepared Ti_3_C_2_T_*x*_ coated cotton fabrics with low Ti_3_C_2_T_*x*_ loading (1.5–2.6 mg cm^−2^) through a facile vacuum filtration process [110]. The fabric with Ti_3_C_2_T_*x*_ loading of 2.6 mg cm^−2^ showed SEE_t_ of 2969 dB cm^2^ g^−1^. Zhang et al. reported that the Ti_3_C_2_T_*x*_ modified fabric with a low Ti_3_C_2_T_*x*_ loading 6 wt% exhibited excellent electrical conductivity of 5 Ω sq^−1^ and outstanding EMI shielding performance (up to 36 dB) [111]. Cheng et al. fabricated a Ti_3_C_2_T_*x*_ MXene-coated cotton fabric by a simple solution impregnation and dip-coating method [112]. The cotton fabric coated by rising amount of Ti_3_C_2_T_*x*_ could improve the EMI shielding performance. When the amount of Ti_3_C_2_T_*x*_ was 5.2 mg cm^−2^, the fabric afforded excellent electrical conductivity of 670.3 S m^−1^ and EMI SE of 31.04 dB in the X-band (Fig. 8a, b). Moreover, the EMI shielding performance of the fabric was almost not changed after 800 bending times (Fig. 8c, d).

#### Polymer

A flexible and durable cellulose/Ti_3_C_2_T_*x*_ MXene nanocomposite fabric was fabricated by a simple dip-coating method [113]. The fabric with a Ti_3_C_2_T_*x*_ nanosheet loading of 1.89 vol% displayed an outstanding electrical conductivity of 2756 S m^−1^ (Fig. 8e). After a polydimethylsiloxane (PDMS) coating, EMI SE of the fabric could achieve over 43 dB in the X and Ku at the Ti_3_C_2_T_*x*_ loading of 1.07 vol%, and no apparent decline was observed after 2000 bending–releasing cycles in the durability test (Fig. 8f).

Zhang’s group fabricated a Ti_3_C_2_T_*x*_ MXene-decorated polyester fabric (Ti_3_C_2_T_*x*_-fabric) by depositing in situ polymerized polypyrrole (PPy)-modified Ti_3_C_2_T_*x*_ nanosheets onto poly (ethylene terephthalate) fabric, followed by silicone coating [114]. The modified fabric displayed a high electrical conductivity of 1000 S m^−1^ and EMI SE of 90 dB, with a thickness of 1.3 mm. Benefiting from the contribution of PPy-to-EM wave absorption due to introduction of the polar group, the EMI shielding performance of the Ti_3_C_2_T_*x*_-fabric was better than that of the fabric modified by MXene at similar conductivities.

Yuan et al. prepared a flexible and stretchable Ti_3_C_2_T_*x*_ MXene/polyurethane (PU) fabric [115]. The Ti_3_C_2_T_*x*_/PU fabric with sandwich structure exhibited EMI SE of ~ 20 dB at a stretching process within 30% deformation.

Polyaniline (PANI)/Ti_3_C_2_T_*x*_/carbon fiber (CF) fabric was fabricated based on the LbL assembly approach [116]. The fabric with a thickness of 0.55 mm possessed a high EMI shielding performance of 26 dB, SSE of 135.5 dB cm^3^ g^−1^ and electrical conductivity of 24.57 S m^−1^.

#### Others

Yu’s group prepared the silk fabric with biomimetic leaf-like MXene/silver nanowire by depositing in situ polymerized PPy-modified Ti_3_C_2_T_*x*_ MXene sheets onto poly (ethylene terephthalate) textiles followed by a silicone coating [117]. The flexible fabric displayed a low sheet resistance of 0.8 Ω sq^−1^, excellent EMI shielding performance of 54 dB in the X-band at the thickness of 120 µm.

Ti_3_C_2_T_*x*_ MXene-decorated wood-pulp fabric was fabricated by depositing highly conductive Ti_3_C_2_T_*x*_ MXene networks onto wood-pulp fabric grid (FG) followed by hydrophobic methyltrimethoxysilane (MTMS) coating with multi-scaled roughness via a simple vacuum-filtration approach and sol–gel process [118]. The fabric possessed superior EMI SE up to ~ 57.8–90.2 dB.

## MXene-Based Materials for EM Wave Absorption

### Pure MXene Matrix

#### Non-annealing

**Fig. 9:**
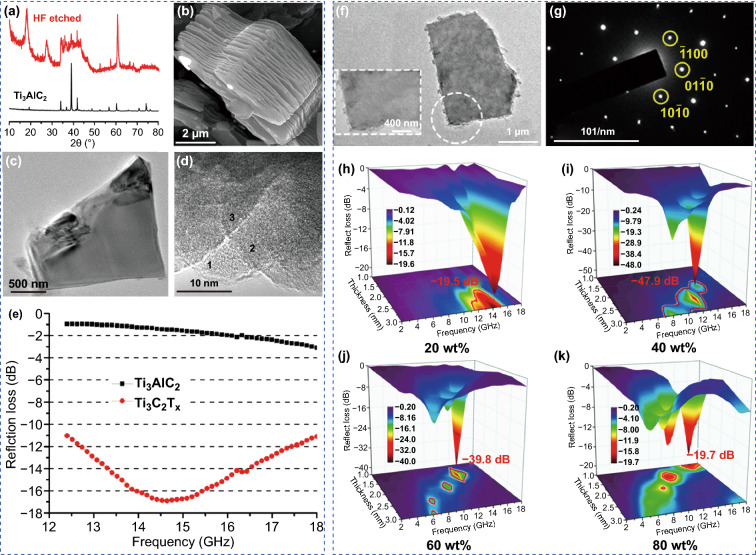
**a** XRD patterns of the Ti_3_AlC_2_ before and after HF treatment at room temperature. **b** SEM image of a multilayer Ti_3_C_2_T_x_ after HF treatment. **c** Transmitted light micrographs of exfoliated Ti_3_C_2_T_x_. **d** TEM images for Ti_3_C_2_T_x_ layers. **e** Thickness dependence of the reflection loss of the 50 wt% Ti_3_C_2_T_x_ filled Ti_3_C_2_T_x_/wax composites in the Ku-band. Reproduced with permission from Ref. [119]. Copyright 2016, Elsevier. **f** High-resolution (HR) TEM image of D-Ti_3_C_2_T_x_. **g** The selected area electron diffraction (SAED) pattern of D-Ti_3_C_2_T_x_. **h–k** Reflection loss of composite with different D-Ti_3_C_2_T_x_/wax weight fraction at different layer thickness. Reproduced with permission from Ref. [44]. Copyright 2019, American Chemical Society

The earliest research was concerned with the EM wave-absorbing properties of multilayer Ti_3_C_2_T_*x*_. Qing et al. prepared multilayer Ti_3_C_2_T_*x*_ MXene by etching Ti_3_AlC_2_ with 50% HF for 3 h (Fig. 9a–d) [119]. Compared with the Ti_3_AlC_2_/wax, the multilayer Ti_3_C_2_T_*x*_/wax showed high EM wave absorption at the same filling concentration of 50 wt% (Fig. 9e). This result was due to the unique two-dimensional (2D) morphology of multilayer Ti_3_C_2_T_*x*_ MXene, such as the large number of defects and larger internal boundary layer capacitance. Feng et al. further confirmed that the excellent EM wave-absorbing property of multilayer Ti_3_C_2_T_*x*_/wax was due to the high dielectric loss and the strong multi-reflections [120]. Luo et al. used a combination of experiment and simulation to study the EM wave absorption of multilayer Ti_3_C_2_T_*x*_ [121], finding that the frequency dispersion effect and the double-peaked dielectric spectral features of Ti_3_C_2_T_*x*_/wax led to superior EM wave absorption.

The influence of different etching time on the EM wave-absorbing performance of multilayer Ti_3_C_2_T_*x*_ has also been studied. Tong et al. investigated the effect of different etching time in 40 wt% HF acid on the EM wave absorption of multilayer Ti_3_C_2_T_*x*_ MXene (0, 6, 24, 48, 96, 144, and 192 h) [122]. With the increase of etching time, the morphology of Ti_3_C_2_T_*x*_ was destroyed gradually. Ti_3_C_2_T_*x*_ etched for 24 h afforded the best EM wave-absorbing properties among etching sample. A minimum RL value of − 42.5 dB was achieved at the thickness of 1.7 mm. This result could be attributed to multiple reflections between MXene layers and interfacial polarizations. Zhao’s group etched the multilayer Ti_3_C_2_T_*x*_ in ≥ 40 wt% HF acid for different time (1, 2, and 3 h) [123] and then explored their EM wave absorption. Multilayer Ti_3_C_2_T_*x*_ etched for 3 h (Ti_3_C_2_T_*x*_-3) had excellent EM wave-absorbing property. Forty percentage ratio multilayer Ti_3_C_2_T_*x*_-3/wax showed a minimum RL value of − 36.3 dB with a thickness of 4.5 mm. Cui et al. produced multilayer Ti_3_C_2_T_*x*_ etched by HCl/LiF with different diverse etching times (12, 24, 36, 48, and 60 h) [124] and studied their EM wave absorbing properties. They found the same result as HF etching. The Ti_3_C_2_T_*x*_ etched 24 h showed best EM wave absorption, owing to multilayer scattering between the laminate structures.

The influence of different etchants on the EM wave-absorbing properties was reported by Xu’s group [125]. Multilayer Ti_3_C_2_T_*x*_ MXenes were obtained by ultrasonication-solvothermal treatment in different solvents including dimethylformamide (DMF), ethanol, and dimethyl sulfoxide (DMSO), respectively. Research showed that multilayer Ti_3_C_2_T_*x*_ treated with DMF showed excellent wave-absorbing properties due to the larger layer space and diminished oxidation effects.

Cao’s group was firstly investigated the EM wave absorption performance of the delaminated Ti_3_C_2_T_*x*_ (d-Ti_3_C_2_T_*x*_) nanosheet etched by HCl/LiF (Fig. 9f, g) [44]. All Ti_3_C_2_T_*x*_ nanosheet/wax composites with different concentrations showed excellent EM wave absorption (Fig. 9h–k). Especially for 40 wt% composite, a minimum RL value of − 47.9 dB and a corresponding absorption bandwidth of 3.6 GHz were achieved at a thickness of 2.5 mm. Moreover, they found the transformation mechanism between EM energy and thermal energy in the composite. The higher the concentration of delaminated Ti_3_C_2_T_*x*_ nanosheet in the composite, more was the conversion of EM energy to thermal energy.

Xu’s group fabricated the multilayer Nb_2_CT_*x*_ MXene by 49 wt% HF etching and solvothermal/hydrothermal treatment [126]. They found that multilayer Nb_2_CT_*x*_ further treated in ethanol showed much more superior absorption capability. This result could be due to the enlarged interlayer spacing, and increased surface functional groups after ethanol-based solvothermal treatment.

#### Annealing

Yin’s group originally reported that the multilayer Ti_3_C_2_T_*x*_ MXene annealed at 800 °C for 2 h in Ar atmosphere had excellent EM wave-absorbing properties [58]. Such excellent EM wave-absorbing properties were due to the surface functional groups of MXene modified by annealing. The annealing led to the formation of a local sandwich structure composed of TiO_2_ nanocrystals and amorphous carbon, which enhanced the EM wave absorption. After that, they investigated the EM wave-absorbing properties of multilayer Ti_3_C_2_T_*x*_ at different annealing temperatures (600, 700, and 800 °C) for 1 h in CO_2_ [127]. The multilayer Ti_3_C_2_T_*x*_ annealed at 800 °C showed best EM wave absorption performance with its RL value achieving − 36 dB and absorption bandwidth of 5.6 GHz. Meanwhile, they explored the EM wave absorption performance of multilayer Ti_3_C_2_T_*x*_ annealed at 500, 800, and 900 °C for 1 h in CO_2_ [128]. The microwave absorption of multilayer Ti_3_C_2_T_*x*_ annealed at 800 °C was best, which was due to the enhanced polarization loss and stronger conduction loss.

Fan et al. fabricated the multilayer Ti_3_C_2_T_*x*_, and annealed it in O_2_ at different temperatures (100, 200, 300, 400, and 500 °C) for 2 h [129]. The multilayer Ti_3_C_2_T_*x*_ calcined at 100 °C showed excellent EM wave-absorbing properties, with a minimum RL value of − 40.07 dB at 19.2 GHz and the absorption bandwidth of 3.8 GHz. This result was attributed to the appropriate complex permittivity and matching impendence.

### MXene Hybrid Matrix

EM wave absorption can be improved by increasing the magnetic loss or dielectric loss. The magnetic loss of the absorber can be improved by doping with magnetic materials. Certain carbon-based materials with high conductivity can be doped into the absorber to enhance the dielectric loss.

#### Magnetic Hybrid

##### Fe-Based

Liu et al. investigated the EM wave absorption of the multilayer Ti_3_C_2_T_*x*_ doped with different concentrations of Fe_3_O_4_ (3, 5, and 10 wt%) (Ti_3_C_2_T_*x*_-3, Ti_3_C_2_T_*x*_-5, Ti_3_C_2_T_*x*_-10) [130]. By tuning the doping concentration of Fe_3_O_4_, the sample showed improved microwave absorption performance. Among them, Ti_3_C_2_T_*x*_-10/wax showed excellent absorption performance, with a maximum RL value of − 57.3 dB. Zhao et al. further investigated the EM wave absorption of multilayer Ti_3_C_2_T_*x*_/Fe_3_O_4_ [131]. The multilayer Ti_3_C_2_T_*x*_/Fe_3_O_4_ exhibited enhanced EM wave absorption compared with pure multilayer Ti_3_C_2_T_*x*_, which is due to the outstanding impedance matching and efficient attenuation. Yang’s group studied the EM wave-absorbing properties of multilayer Ti_3_C_2_T_*x*_ doped with Fe_3_O_4_ nanoparticles (Fe_3_O_4_@Ti_3_C_2_T_*x*_) with different concentrations [132]. The sample contained 25 wt% Fe_3_O_4_ nanoparticle displayed outstanding EM wave absorption, with a minimum RL value of − 57.2 dB at 15.7 GHz and bandwidth of 1.4 GHz, caused by enhanced interface polarization. Che’s group fabricated magnetized multilayer Ti_3_C_2_T_*x*_ MXene microsphere by embedded Ti_3_C_2_T_*x*_ MXene into a confined and magnetized Fe_3_O_4_ nanospheres (designated as M/F) [133]. This structure could enhance the specific interfaces and dielectric polarization. Meanwhile, these Fe_3_O_4_ magnetic led to the optimized impedance balance and EM coordination capability. As expected, the M/F composite with 15 wt% Fe_3_O_4_ content hold distinct EM wave absorption property with the strong reflection loss (− 50.6 dB) and absorption bandwidth (4.67 GHz) at the thickness of 2 mm.

The multilayer Ti_3_C_2_T_*x*_/flaky carbonyl iron (FCI) composite with different mass ratios were fabricated by the ultrasonic mixing method [134]. An excellent EM wave-absorbing properties can be realized by optimizing the Ti_3_C_2_T_*x*_ and FCI content. Beneficial from the good impedance matching and moderate attenuation ability, the composite with 20 wt% Ti_3_C_2_T_*x*_ and 40 wt% FCI loading presented the absorption bandwidth of 8.16 GHz with a thickness of 1.0 mm.

##### Ni-Based

A Ti_3_C_2_T_*x*_/Ni-nanoparticle hybrid was synthesized by in situ hydrothermal treatment. The Ti_3_C_2_T_*x*_-Ni hybrid showed a *RL* value of − 47.06 dB with a thickness of 1.5 mm and bandwidth of 3.6 GHz [135]. The combined effect of magnetic loss, conduction loss and dielectric loss is the key to achieving such excellent EM-absorbing ability. Liang et al. prepared a Ti_3_C_2_T_*x*_ MXene/Ni-nanochain (Ni@MXene) hybrid via a facile and moderate co-solvothermal method [36]. The Ni@MXene hybrid displayed a minimum *RL* of − 49.9 dB at the thickness of 1.75 mm when the Ni-nanochain content was 90 wt%. It was further proved that the synergistic effect of conductive MXene and the magnetic Ni-nanochain led to the excellent EM wave-absorbing ability. Che’s group also investigated the EM wave-absorbing properties of a multilayer Ti_3_C_2_T_*x*_/Ni hybrid [136]. The Ni nanoparticles were uniformly distributed on the surface and in the multilayered gaps of Ti_3_C_2_T_*x*_. This unique structure led to excellent EM wave-absorbing properties. The hybrid showed a minimum *RL* of − 50.5 dB at 5.5 GHz. Liang et al. investigated the EM wave-absorbing properties of Ni-, Co- and NiCo-doped multilayer Ti_3_C_2_T_*x*_ [137]. Among them, multilayer Ti_3_C_2_T_*x*_ doped with Ni nanoparticles (Ni@Ti_3_C_2_T_*x*_) in a polyvinylidene fluoride (PVDF) matrix showed strong EM wave absorption. With 10 wt% Ni dopant, the sample exhibited the optimal EM wave absorption, with a minimum *RL* of − 52.6 dB at 8.4 GHz and absorption bandwidth of 3.7 GHz. Liu et al. found that compared with the individual Ti_3_C_2_T_*x*_ and Ni powders [138], hybrid Ti_3_C_2_T_*x*_/Ni afforded the most favorable EM wave absorption performance with a minimum RL value of − 24.3 dB at 9.8 GHz.

##### Co-Based

Deng et al. fabricated the Co_3_O_4_/Ti_3_C_2_T_*x*_ by the two-step method. When the mass ratio of Ti_3_C_2_T_*x*_ to Co_3_O_4_ was 1:3 [139], the Co_3_O_4_/Ti_3_C_2_T_*x*_ hybrid showed better than 90% absorption from 10.8 to 17 GHz. Such excellent performance is owing to combined effects of multilayer structure, defects, conductivity of Ti_3_C_2_T_*x*_ and equivalent capacitance of Co_3_O_4_.

##### Multi-based

A CoFe@Ti_3_C_2_T_*x*_ hybrid was prepared by in situ reduction, and a minimum *RL* value of − 36.29 dB could be obtained with a thickness of 2.2 mm [140]. The excellent EM wave absorption performance was due to the sandwich-like structure and enhanced interfacial polarization. He et al. further confirmed that multilayer Ti_3_C_2_T_*x*_ modified by CoFe could improve the EM wave absorption.

A FeCo@Ti_3_C_2_T_*x*_ hybrid was fabricated by in situ hydrothermal treatment [141]. The incorporation of magnetic FeCo could improve the EM wave-absorbing property. The FeCo@Ti_3_C_2_T_*x*_ hybrid exhibited a broad EM wave-absorbing bandwidth of 8.8 GHz, due to enhanced impedance matching and microwave attenuation.

A Co-doped NiZn ferrite (CNZF)/polyaniline (PANI) on Ti_3_C_2_T_*x*_ hybrid (CNZF/PANI/Ti_3_C_2_T_*x*_) was synthesized by hydrothermal reaction and interfacial polymerization [142]. The dipole polarization, interfacial polarization, natural resonance, eddy current loss, and multiple reflections contributed to the improved EM wave absorption performance of CNZFO/PANI/Ti_3_C_2_T_*x*_ hybrid. The multiple-layer hybrid exhibited excellent EM wave absorption with a minimum *RL* of − 37.1 dB and absorption bandwidth of 4.1 GHz.

Hou et al. investigated the EM wave-absorbing properties of NiCo_2_O_4_-doped multilayer Ti_3_C_2_T_*x*_ at different annealing temperatures (350, 400, 450, and 500 °C) for 2 h in argon [143]. The NiCo_2_O_4_/Ti_3_C_2_T_*x*_ annealed at 350 °C displayed the best EM wave absorption, with the *RL* value of − 50.96 dB. This result was attributed to the polarization behavior and multiple scattering produced by unique structures.

The EM wave-absorbing properties of Ba_3_Co_2_Fe_24_O_41_, multilayer Ti_3_C_2_T_*x*_, and polyvinyl butyral (PVB) after physical mixing were investigated by Yang’s group [144]. The as-synthesized PVB/Ba_3_Co_2_Fe_24_O_41_/Ti_3_C_2_T_*x*_ exhibited outstanding and efficient EM wave attenuation. A minimum RL value for the PVB/Ba_3_Co_2_Fe_24_O_41_/Ti_3_C_2_T_*x*_ composite reached − 46.3 dB at 5.8 GHz; the absorption bandwidth was 1.6 GHz, with a thickness of only 2.8 mm.

#### Carbon-Based Hybrid

**Fig. 10:**
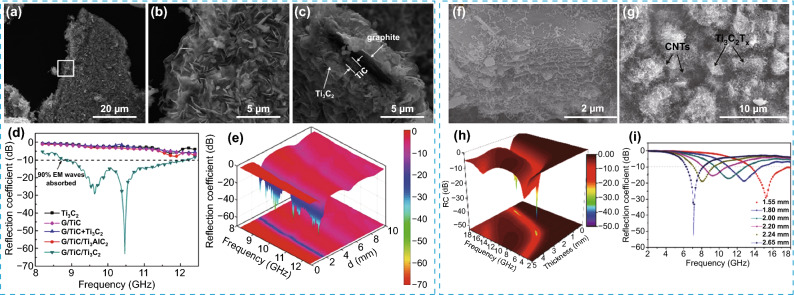
SEM image of **a-c** G/TiC/Ti_3_C_2_T_x_. **d** RC of G/TiC/Ti_3_C_2_T_x_, Ti_3_C_2_T_x_, G/TiC/Ti_3_AlC_2_, G/TiC, and G/TiC + Ti_3_C_2_T_x_ in paraffin matrix with the loading of 50 wt% with the sample thickness of 2.1 mm. **e** 3D RC of G/TiC/Ti_3_C_2_T_x_ versus frequency and sample thickness. Reproduced with permission from Ref. [145]. Copyright 2018, WILEY–VCH. SEM images of **f** and **g** Ti_3_C_2_T_x_/CNT. **h** 3D representations and **i** theoretical curves of RC versus frequency and thickness of Ti_3_C_2_T_x_/CNT with a filler loading of 35 wt%. Reproduced with permission form Ref. [146]. Copyright 2017, The Royal Society of Chemistry

A graphite/TiC/Ti_3_C_2_T_*x*_ (G/TiC/Ti_3_C_2_T_*x*_) hybrid was obtained by two steps [145]. Firstly, the graphite/TiC/Ti_3_AlC_2_ (G/TiC/Ti_3_AlC_2_) hybrid was prepared in a bath of molten salts. G/TiC/Ti_3_C_2_T_*x*_ was obtained after etching the Al atoms from G/TiC/Ti_3_AlC_2_ (Fig. 10a–c). It was found that the graphite/TiC/Ti_3_AlC_2_-wax matrix with a thickness of 2.1 mm exhibited a minimum *RL* of − 63 dB and the effective absorption bandwidth was more than 3.5 GHz (Fig. 10d, e).

Multilayer Ti_3_C_2_T_*x*_ MXene modified with in situ grown carbon nanotubes (Ti_3_C_2_T_*x*_/CNT) was fabricated by Yin’s group (Fig. 10f, g) [146]. Compared with the pure multilayer Ti_3_C_2_T_*x*_ MXenes, the hierarchical microstructure makes a contribution to the outstanding EM wave absorption performance, with a minimum *RL* value of − 52.9 dB and absorption bandwidth of 4.46 GHz (Fig. 10h, i).

Dai et al. reported that the Ti_3_C_2_T_*x*_ MXenes/nano-carbon sphere hybrid exhibited *RL* of − 54.67 dB at 3.97 GHz [147], owing to the unique structure of Ti_3_C_2_T_*x*_ MXenes/nano-carbon and the formation of a heterogeneous interface structure.

Nitrogen-doped graphene (N-GP) and Ti_3_C_2_T_*x*_ composites were prepared by Qing group [148]. The values and frequency dependencies of EM properties of N-GP/Ti_3_C_2_T_*x*_ could be tuned by the combination of the unique structure and dielectric characteristics of the N-GP and Ti_3_C_2_T_*x*_. A minimum RL of the N-GP/Ti_3_C_2_T_*x*_ composite reached up to − 52 dB, and absorption bandwidth could be obtained in the frequency range of 10.9–18 GHz with a thickness of only 1.4 mm.

#### Others Hybrid

Qian et al. prepared an urchin-like ZnO–Ti_3_C_2_T_*x*_ hybrid through a coprecipitation process [149]. Compared with pure multilayer Ti_3_C_2_T_*x*_, ZnO–Ti_3_C_2_T_*x*_ hybrid showed significant enhanced EM wave absorption. The minimum RL of 75 wt% ZnO–Ti_3_C_2_T_*x*_/wax realized − 26.30 dB, which is much better than that of pure multilayer Ti_3_C_2_T_*x*_ (− 6.70 dB), owing to larger interfaces and the construction of semiconductive networks.

Ti_3_C_2_T_*x*_ MXenes/polypyrrole microspheres (Ti_3_C_2_T_*x*_/PPy) composites with delaminated structure were fabricated for significant enhancement of EM wave-absorbing properties [150]. Thanks to the synergistic effect between Ti_3_C_2_T_*x*_ and PPy microspheres, the obtained Ti_3_C_2_T_*x*_@PPy composite exhibited excellent EM absorption performance. The 10 wt% Ti_3_C_2_T_*x*_@PPy composites in wax matrix displayed a minimum RL of − 49.5 dB at 7.6 GHz, and the absorption bandwidth was 5.14 GHz. Tong et al. reported that multilayer Ti_3_C_2_T_*x*_ MXene decorated with PPy chains is a good microwave-absorbing material [151]. The 25 wt% Ti_3_C_2_T_*x*_/PPy hybrids in a wax matrix showed a minimum *RL* of − 49.2 dB.

A multilayer Ti_3_C_2_T_*x*_ modified by MoS_2_ was fabricated by a hydrothermal method [152]. The complex permittivity of MoS_2_/Ti_3_C_2_T_*x*_ increased compared with that of multilayer Ti_3_C_2_T_*x*_. This result led to enhanced EM wave-absorbing performance. When the thickness of MoS_2_/Ti_3_C_2_T_*x*_-wax was 2.5 mm, the corresponding absorption bandwidth was 2.6 GHz.

A multilayer Ti_3_C_2_T_*x*_ MXene/polyaniline (PANI) was prepared by the hydrothermal reaction [153]. The EM wave absorption of sample with different PANI doping concentrations was systematically studied. When the mass ratio of Ti_3_C_2_T_*x*_ to polyaniline is 1:2, the sample showed best EM wave absorption. The maximum *RL* reached − 56.30 dB at 13.80 GHz with a thickness of 1.8 mm.

The Nb_2_O_5_ with different morphologies was prepared in situ and implanted between the layers of the Nb_2_CT_*x*_ MXene via hydrothermal method [154]. The Nb_2_O_5_/Nb_2_CT_*x*_ exhibited the enhanced EM wave absorption compared with primary Nb_2_CT_*x*_ MXene. Especially, Nb_2_CT_*x*_ doped with columnar Nb_2_O_5_ showed a minimum *RL* of − 44.1 dB at 2.8 GHz, owing to the increased lamellar spacing of the Nb_2_CT_*x*_.

### Foam

**Fig. 11:**
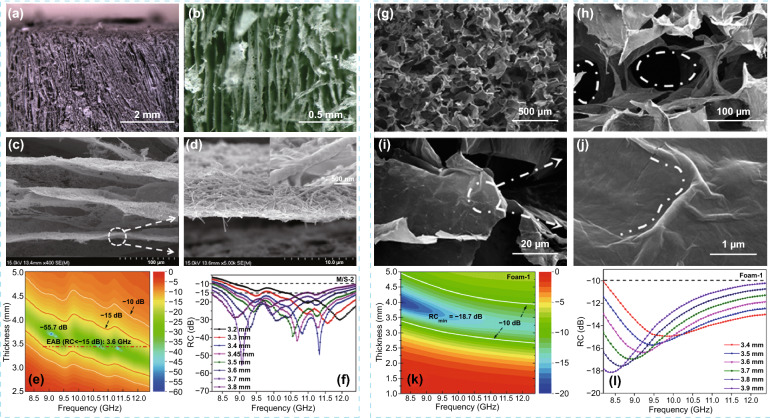
**a, b** Digital photographs of f-Ti_2_CT_x_/SiCnws hybrid foams. **c, d** Typical cross-sectional SEM images of f-Ti_2_CT_x_/SiCnws hybrid layers. 2D contours (**e)** and RC curves (**f**) vs. frequency (8.2–12.4 GHz) and thickness (0–5 mm) of f-Ti_2_CT_x_/SiCnws hybrid foam. Reproduced with permission from Ref. [156]. Copyright 2018, American Chemical Society. **g-j** Typical images of f-Ti_2_CT_x_/PVA foam. 2D contours **k** and reflection coefficient **l** curves versus frequency and thickness of f-Ti_2_CT_x_/PVA foams. Reproduced with permission from Ref. [100]. Copyright 2019, American Chemical Society

Yin’s group fabricated reduced graphene oxide (RGO)/Ti_3_C_2_T_*x*_ hybrids foam via self-assembly and sacrificial template processes [155]. The RGO/Ti_3_C_2_T_*x*_ foam with the density is merely 0.0033 g cm^−3^ possessed outstanding EM absorption performance superior to all reported foam-based counterparts, and the absorption bandwidth covers the whole X-band at 3.2 mm, and its specific EM absorption performance value exceeds 14,299.2 dB cm^−2^ g^−1^. Those results were attributed to the unique heterogeneous interface associated with core–shell structures. Meanwhile, they synthesized ordered lamellar few-layered Ti_3_C_2_T_*x*_/SiC nanowires (f-Ti_3_C_2_T_*x*_/SiCnws) hybrid foams with ultralow density via a combination of self-assembly and bidirectional freezing processes (Fig. 11a–d) [156]. The f-Ti_3_C_2_T_*x*_/SiCnws hybrid foam showed a minimum *RL* of − 55.7 dB at an ultralow density of only about 0.029 g cm^−3^ (Fig. 11e, f). Compared with most of the current foam-based counterparts, the free-standing foams exhibited enhanced EM absorption properties, owing to enhanced polarization loss and balance the conductive loss and impedance matching characteristics caused by the unique microstructure and phase compositions. After that, they prepared porous Ti_2_CT_*x*_ MXene/poly vinyl alcohol (PVA) composite foams constructed by a facile freeze-drying method (Fig. 11g–j) [100]. Ti_2_CT_*x*_/PVA foam possessed an outstanding EM absorption performance with a minimum *RL* of − 18.7 dB and an absorption covering the whole X-band with any thickness from 3.4 to 3.9 mm (Fig. 11k, l).

A new ultralight carbon foam modified by Ti_3_C_2_T_*x*_ nanosheet (CF/Ti_3_C_2_T_*x*_) with three-dimensional network structure was prepared by vacuum impregnation and freeze-drying process [157]. The CF/Ti_3_C_2_T_*x*_ foam with ultralow density of only 5–7 mg cm^−3^ showed excellent flexibility and steady compression-resilience properties. Meanwhile, the foam showed higher EM absorption than most foam-based EM absorbers, with a minimum *RL* of − 45 dB at 8.8 GHz.

### Aerogel

Yang et al. prepared aligned Ti_3_C_2_T_*x*_ MXene/gelatin (M@G) nanocomposite aerogel using a unidirectional freeze casting method [158]. The composite aerogel showed a minimum RL of − 59.5 dB at 14.04 GHz and an absorption bandwidth of 6.24 GHz in the parallel direction but presented a minimum *RL* of − 57.3 dB at 4.08 GHz with an absorption bandwidth covering of 3.72–4.56 GHz in the vertical direction. The aerogel exhibited significantly anisotropic EM wave-absorbing properties, owing to the unidirectional aligned microstructure.

Jiang et al. fabricated the hierarchically structured cellulose aerogels with interconnected Ti_3_C_2_T_*x*_ MXene nanosheet networks via a freeze-casting and chemical cross-linking strategy [159]. The aerogel with a low density (0.31 g cm^−3^) presented a minimum RL of − 43.4 dB at 11.2 GHz and an absorption bandwidth of 4.5 GHz, which was due to enhanced conductive loss and multiple reflection attenuating.

A TiO_2_/Ti_3_C_2_T_*x*_/RGO ternary composite aerogel with a three-dimensional hierarchical architecture was synthesized by a hydrothermal method [160]. The minimum RL of the aerogel reached − 65.3 dB at the thickness of 2.5 mm. At the same time, the absorption bandwidth was 4.3 GHz, with a thickness of only 2.0 mm. The improved EM wave absorption performance was attributed to the highly porous conductive networks, multiple reflection, and scattering and defective polarization properties.

Wang et al. reported a multilayer Ti_3_C_2_T_*x*_@RGO hybrid aerogel prepared by a hydrothermal method and freeze-drying treatment [161]. Compared with pure multilayer Ti_3_C_2_T_*x*_, the EM wave absorption of the Ti_3_C_2_T_*x*_@RGO aerogel improved significantly. The minimum RL achieved for the Ti_3_C_2_T_*x*_@RGO aerogel was − 31.2 dB at 8.2 GHz, and the absorption bandwidth reached 5.4 GHz. Such good performance was due to the conductive network, interface polarization, dipole polarization, and multiple scattering, as important contributors.

### Fabric

To obtain the enhanced EM wave absorption performance, the hierarchical Ti_3_C_2_T_*x*_ MXene/Ni chain/ZnO array hybrid nanostructures were rationally constructed on cotton fabric [162]. The impedance matching could be modulated by adjusting the concentration of Ni chains to manipulate the magnetic loss. The minimum *RL* value for the hybrid fabric could reach − 35.1 dB at 8.3 GHz at the thickness of 2.8 mm, and its absorption bandwidth could cover the whole X-band with thickness of 2.2 mm.

## Overview and Perspectives

**Fig. 12:**
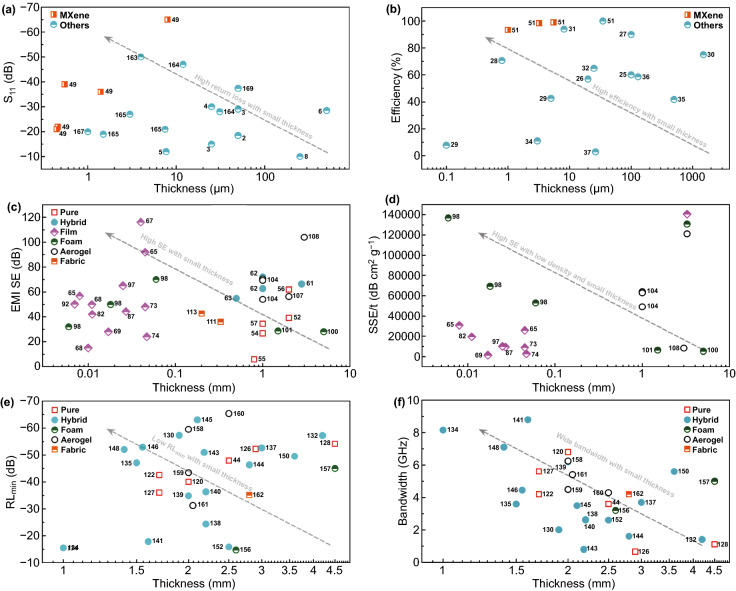
Comparison of **a** the return loss and **b** efficiency of MXene antenna with other materials. **c** EMI SE and **d** SEE_t_ versus thickness for MXene-based shielding materials. **e** RL_min_ and **f** absorption bandwidth versus thickness of typical MXene-based absorbing materials

As shown in Table 1, MXene antennas exhibit excellent comprehensive performance compared with other materials [163–169], as clearly confirmed by Fig. 12a, b. This indicates that MXene antennas have great development prospects for the future. Research on the EM attenuation properties of MXenes was initiated in 2016. Thereafter, a large number of MXene-based structures have been designed for EMI shielding and EM wave absorption based on the principle of “lightweight, wide, and strong.” Tables 2 and 3 list the EMI shielding and EM wave absorption performances of MXene-based materials, respectively. Research on MXene-based shielding and absorption materials has mainly focused on film and hybrid materials (Fig. 12c–f), respectively. MXene-based films afford the highest conductivity among this class of materials (Table 2), which is the reason why MXene films are the most widely used materials for EMI shielding. However, the high conductivity leads to impedance mismatch. Therefore, no research has been performed on MXene films for EM wave absorption (Table 3). MXene hybrids for EM wave absorption are the most extensively studied materials, owing to their easily tunable EM parameters. Moreover, the addition of a matrix also simplifies adjustment for impedance matching. The choice of foam and aerogel is based on their lightweight characteristics (Fig. 12d), and the porous structure can increase multiple reflections to improve the attenuation of EM waves. The advantage of using a fabric as the EM attenuation material is that it has satisfactory porosity and permeability. In addition, it is more practical.

**Table 1:** The comprehensive performance of patch antennas made of different materials

Type	*d* (µm)	Efficiency (100%)	Frequency (GHz)	Substrate	Substrate thickness (mm)	*σ* (S m^−1^)	References
Ti_3_C_2_T_*x*_ MXene	1	80–93.4	5.6, 10.9, and 16.4	RT 5880	1.6	1.5 × 10^6^	[51]
	3.2	87–98.4					
	5.5	90.6–99					
Copper	35	95–100		RT 5880	1.6	5.8 × 10^7^	[51]
EGaIn	100	46–60	3.45	PDMS	1	3.4 × 10^6^	[25]
Copper mesh	20	49–56.88	2.4–2.5	acrylic	1.2	1 × 10^6^	[26]
SWCNTS/EGaIn	100	90	4	PDMS	0.5	3.4 × 10^6^	[27]
NbSe_2_	0.8	70.6	2.01–2.8	PET	–	9.7 × 10^5^	[28]
IZTO/Ag/IZTO	0.1	7.76	2.45	Acryl	1	–	[29]
Cu mesh	5	42.69	2.45	Acryl	1	–	[29]
EGaIn	1500	75	5.2	Photopolymer resin	6	5.1 × 10^6^	[30]
Aligned CNTs	8.16	94	10, 14	RT 5870	–	4.63 × 10^6^	[31]
Graphene	25	64.9	6	PDMS	2	–	[32]
Graphene/CNTs/PMMA	–	44.9	3.11	PET	–	–	[33]
Silver	3	11	2.45	Cardboard	0.56	2 × 10^7^	[34]
Silver nanowire	500	41.83	2.92	PDMS	1	8.1 × 10^5^	[35]
Ni/Ag/Cu fabric	130	58.6	2.45	PDMS	3	–	[36]
Silver paint	26.5	2.8	2.45	NinjaFlex	1.2	1.7 × 10^4^	[37]

**Table 2:** Typical MXene-based materials and their EMI shielding properties

Type	Materials	Matrix	Ratio (wt%)	*d* (mm)	*σ* (S m^−1^)	SE (dB)	SEE_t_ (dB cm^2^ g^−1^)	References
Pure MXene	Ti_3_C_2_T_*x*_	Wax	60	2	–	39.1	–	[52]
	Ti_3_C_2_T_*x*_	SiO_2_	60	1	0.42	26.7	–	[54]
	Ti_2_CT_*x*_	Wax	40	0.8	1.63 × 10^–16^	6	–	[55]
	Ti_3_C_2_T_*x*_	PS	1.9 vol%	2	1081	62	–	[56]
	Ti_3_C_2_T_*x*_	PVDF	50	1	0.988	34.49	–	[57]
MXene hybrid	Ti_3_C_2_T_*x*_–Ni	Wax	50	2.8	4	66.4	–	[61]
	Ti_3_C_2_T_*x*_–Ag	Wax	60	1	3.813	62.7		[62]
	Nb_2_CT_*x*_–Ag	Wax	60	1	3.123	72.04	–	[62]
	r GO–Ti_3_C_2_T_*x*_	Epoxy	4.5	0.5	387.1	55	–	[63]
MXene film	Ti_3_C_2_T_*x*_	–	100	0.045	4.665 × 10^5^	92	25,863	[65]
	Ti_3_C_2_T_*x*_	–	100	5.5 × 10^−5^	5 × 10^5^	20	3.89 × 10^6^	[66]
	Ti_3_CNT_*x*_	–	100	0.04	1.786 × 10^5^	116.2	–	[67]
	Ti_2_CT_*x*_	–	100	0.011	1.6 × 10^5^	50	–	[68]
	Nb_2_CT_*x*_	–	100	0.01	500	15	–	[68]
	Ti_3_C_2_T_*x*_–SA	–	90	0.008	2.9 × 10^5^	57	30,830	[65]
	Ti_3_C_2_T_*x*_/ANF	–	80	0.017	1.733 × 10^4^	28	1317.64	[69]
	ANF/Ti_3_C_2_T_*x*_/Ag	–	20	0.045	9.22 × 10^4^	48.1	8907.4	[73]
	Ti_3_C_2_T_*x*_/CNF	–	90	0.047	739.4	24	2647	[74]
	Ti_3_C_2_T_*x*_/PEDOT:PSS	–	87.5	0.0111	3.405 × 10^4^	42.10	19,497.8	[82]
	Ti_3_C_2_T_*x*_/PVA	–	19.5	0.027	716	44.4	9343	[87]
	Ti_3_C_2_T_*x*_/GO	–	90	0.007	2.64 × 10^5^	50.2	–	[92]
	Ti_3_C_2_T_*x*_/MMT	–	90	0.025	4420	65	10,000	[97]
MXene foam	Ti_3_C_2_T_*x*_	–	100	0.006	58,820	32	136,752	[98]
	Ti_2_CT_*x*_/PVA	–	0.15 vol%	5	8.3 × 10^–6^	28	5136	[100]
	Ti_3_C_2_T_*x*_/rGO	–	33	1.5	1000	28.6	6217	[101]
MXene aerogel	Ti_3_C_2_T_*x*_	–	100	1	–	70.5	64,182	[104]
	Ti_2_CT_*x*_	–	100	1	–	69.2	62,909	[104]
	Ti_3_CNT_*x*_	–	100	1	–	54.1	49,182	[104]
	Ti_3_C_2_T_*x*_/rGO	Epoxy	0.99 vol%	2	695.9	56.4	–	[107]
	Ti_3_C_2_T_*x*_/CNT	–	25	3	943	103.99	8253.17	[108]
MXene fabric	Ti_3_C_2_T_*x*_	Cotton	6	0.33	5 Ω sq^−1^	36	–	[111]
	Ti_3_C_2_T_*x*_	Cotton	5.2 mg/cm^2^	–	670.3	31.04	–	[112]
	Ti_3_C_2_T_*x*_	Cellulose	1.89 vol%	0.2	2756	42.7	–	[113]

**Table 3:** Typical MXene-based materials and their EM wave absorption properties

Type	Materials	Matrix	Ratio (wt%)	*d* (mm)	RL_min_ (dB)	Bandwidth (< − 10 dB) (GHz)	References
Pure MXene	Ti_3_C_2_T_*x*_	Wax	40	2.5	− 47.9	3.6	[44]
	Ti_3_C_2_T_*x*_	Wax	50	2	− 40	6.8	[120]
	Ti_3_C_2_T_*x*_	Wax	55	1.7	− 42.5	4.2	[122]
	Nb_2_CT_*x*_	Wax	70	2.9	− 52.2	0.65	[126]
	Ti_3_C_2_T_*x*_	Wax	45	1.7	− 36	5.6	[127]
	Ti_2_CT_*x*_	Wax	55	4.5	− 54.1	1.1	[128]
MXene hybrid	TiO_2_/Ti_3_C_2_T_*x*_/Fe_3_O_4_	Wax	70	1.9	− 57.3	2	[130]
	Fe_3_O_4_@Ti_3_C_2_T_*x*_	Wax	60	4.2	− 57.2	1.4	[132]
	Ti_3_C_2_T_*x*_/FCI	Epoxy	60	1	− 15.52	8.16	[134]
	Ti_3_C_2_T_*x*_/Ni	Wax	50	1.5	− 47.06	3.6	[135]
	Ni@Ti_3_C_2_T_*x*_	PVDF	10	3	− 52.6	3.7	[137]
	Ti_3_C_2_T_*x*_/Ni	Wax	60	2.2	− 24.3	2.6	[138]
	Ti_3_C_2_T_*x*_/Co_3_O_4_	Wax	50	2	− 34.8	6.2	[139]
	CoFe@Ti_3_C_2_T_*x*_	Wax	60	2.2	− 36.29	2.64	[140]
	FeCo@Ti_3_C_2_T_*x*_	Wax	70	1.6	− 17.86	8.8	[141]
	Ti_3_C_2_T_*x*_–NiCo_2_O_4_	Wax	50	2.18	− 50.96	0.8	[143]
	Co_2_Z/Ti_3_C_2_T_*x*_	PVB	30	2.8	− 46.3	1.6	[144]
	G/TiC/Ti_3_C_2_T_*x*_	Wax	50	2.1	− 63	3.5	[145]
	CNT/Ti_3_C_2_T_*x*_	Wax	35	1.55	− 52.9	4.46	[146]
	N-GP/Ti_3_C_2_T_*x*_	Epoxy/PA	32	1.4	− 52	7.1	[148]
	Ti_3_C_2_T_*x*_@PPy	Wax	10	3.6	− 49.5	5.6	[150]
	MoS_2_@TiO_2_/Ti_3_C_2_T_*x*_	Wax	50	2.5	− 15.9	2.6	[152]
MXene foam	Ti_3_C_2_T_*x*_/SiC	–	100	2.6	− 14.7	3.2	[156]
	CF/Ti_3_C_2_T_*x*_	Wax	–	4.5	− 45	5	[157]
MXene aerogel	Ti_3_C_2_T_*x*_@gelatin	–	100	2	− 59.5	6.24	[158]
	Ti_3_C_2_T_*x*_/Cellulose	Wax	24	2	− 43.4	4.5	[159]
	TiO_2_/Ti_3_C_2_T_*x*_/RGO	Wax	10	2.5	− 65.3	4.3	[160]
	Ti_3_C_2_T_*x*_@RGO	Wax	15	2.05	− 31.2	5.4	[161]
MXene fabric	Ti_3_C_2_T_*x*_/Ni/ZnO	Cotton	13.39	2.8	− 35.1	4.2	[162]

To summarize, the core outstanding areas to be addressed in the wireless communication and EM attenuation fields include the following: conduction design, because the conduction directly affects the thickness and transmission performance of the antenna, the strength of the first interface reflection is dominated by conduction, and conduction loss plays an important role in EM wave absorption; EM transmission and attenuation mechanisms in MXenes that are not understood and may fundamentally differ from the behavior in other 2D materials, such as graphene; how to balance EM wave absorption and reflection in MXenes to achieve green shielding, and how wireless communication devices employing MXenes can be adapted to large-scale industrial production in the future.

Based on the above overview of MXenes, there are still some challenges that need to be addressed in the future. The conduction is mainly decided by the type of MXene, number and types of surface functional groups, and the construction mode of MXenes. Conduction varies greatly in different kinds of MXenes. For example, the conductivity of a free-standing Mo_2_Ti_2_C_3_ film is 100 S cm^−1^, while the conductivity of a free-standing Ta_4_C_3_ film is 0.476 S cm^−1^ [24]. However, current research has mainly focused on Ti_3_C_2_T_*x*_ MXene. It is necessary to study the wireless communication performance and EM response mechanism of other MXenes. The number and types of surface functional groups are mainly affected by the etching method. For example, Ti_3_C_2_T_*x*_ etched with LiF/HCl has a high content of = O terminal groups compared with Ti_3_C_2_T_*x*_ etched with HF, which leads to conductivity differences [54]. The annealing temperature also has a certain influence on the type of surface functional groups [24]. Researchers need to study how to control the conductivity of MXenes by selecting different etchants and annealing temperatures. Moreover, the method used to construct MXenes also affects the conductivity of MXenes. For example, the conductivity of a pressed Ti_3_C_2_T_*x*_ disk (2 S cm^−1^) is lower than that of a free-standing Ti_3_C_2_T_*x*_ film (1500 S cm^−1^). Designing different methods of constructing MXenes to regulate the conduction will also be a direction for future research. The oxidation of MXenes is an unavoidable problem, especially in the case of few-layer or monolayer MXenes. After oxidation, the conductivity of MXenes decreases significantly. How to inhibit the oxidation of MXenes is a key point for future research.

At present, research on the wireless communication performance of MXenes is still in the exploratory stage. The mechanism of transmission of EM waves in MXene antennas is not very clear. Although research on EM attenuation in MXenes is more extensive, the exact mechanism of EMI shielding and EM wave absorption is still poorly understood. Since the concept of “green shielding” was put forward, realizing green shielding by using MXene-based materials has become a major challenge. Therefore, a series of thorough studies on EM wave transmission, dielectric relaxation, and the EM response of MXene-based materials is urgently needed.

The low yield of MXenes limits their commercial application in wireless communication, EMI shielding, and EM wave absorption materials. Increasing the production of MXenes is the only way to realize commercialization. Moreover, the hydrophilic nature of MXenes limits the fabrication of composites or hybrids with polymers and other materials to only aqueous media. To broaden the path of structural design, it is essential to investigate organic dispersions of MXenes.

In summary, MXenes, as the newest and fastest growing family of 2D materials, will open new avenues for realizing various classes of wireless communication and EM protection devices. This review is expected to serve as a guide to those exploring wireless communication and EM attenuation properties of MXenes.
